# Identification of a new alanine racemase in *Salmonella* Enteritidis and its contribution to pathogenesis

**DOI:** 10.1186/s13099-018-0257-6

**Published:** 2018-07-10

**Authors:** Shilpa Ray, Susmita Das, Pritam Kumar Panda, Mrutyunjay Suar

**Affiliations:** School of Biotechnology, KIIT University, Bhubaneswar, Odisha India

**Keywords:** *Salmonella* Enteritidis, Alanine racemase, d-Alanine auxotrophy, Alr, SEN3897, Inflammation, Kinetic parameters

## Abstract

**Background:**

Non-typhoidal *Salmonella* (NTS) infections caused primarily by *S.* Enteritidis and *S.* Typhimurium particularly in immunocompromised hosts have accounted for a large percentage of fatalities in developed nations. Antibiotics have revolutionized the cure of enteric infections but have also led to the rapid emergence of pathogen resistance. New powerful therapeutics involving metabolic enzymes are expected to be potential targets for combating microbial infections and ensuring effective health management. Therefore, the need for new antimicrobials to fight such health emergencies is paramount. Enteric bacteria successfully evade the gut and colonize their hosts through specialized virulence strategies. An important player, alanine racemase is a key enzyme facilitating bacterial survival.

**Results:**

This study aims at understanding the contribution of alanine racemase genes *alr*, *dadX* and *SEN3897* to *Salmonella* survival in vitro and in vivo. We have shown *SEN3897* to function as a unique alanine racemase in *S*. Enteritidis which displayed essential alanine racemase activity. Interestingly, the sole presence of this gene in *alr dadX* double mutant showed a strict dependence on d-alanine supplementation both in vitro and in vivo. However, Alr complementation in d-alanine auxotrophic strain restored the alanine racemase deficiency. The K_m_ and V_max_ of SEN3897 was 89.15 ± 10.2 mM, 400 ± 25.6 µmol/(min mg) for l-alanine and 35 ± 6 mM, 132.5 ± 11.3 µmol/(min mg) for d-alanine, respectively. In vitro assays for invasion and survival as well as in vivo virulence assays involving SEN*3897* mutant showed attenuated phenotypes. Further, this study also showed attenuation of d-alanine auxotrophic strain in vivo for the development of potential targets against *Salmonella* that can be investigated further.

**Conclusion:**

This study identified a third alanine racemase gene unique in *S*. Enteritidis which had a potential effect on survival and pathogenesis in vitro and in vivo. Our results also confirmed that SEN3897 by itself wasn’t able to rescue d-alanine auxotrophy in *S*. Enteritidis which further contributed to its virulence properties.

**Electronic supplementary material:**

The online version of this article (10.1186/s13099-018-0257-6) contains supplementary material, which is available to authorized users.

## Background

Salmonellosis is one of the leading causes of mortality among infectious diseases [1]. *Salmonella enterica* are Gram-negative facultative intracellular pathogens bearing around 2400 serovars which either have certain host specificity or infect a huge variety of hosts [2]. Annually nearly a million deaths are attributed to enteric pathogens; henceforth the health concerns rise for the development of targets for drug design [3]. *Salmonella* consists of two species namely *S. bongori* and *S. enterica*. The two epidemiologically significant serovars are *Salmonella enterica* serovar Typhimurium (*S*. Typhimurium) and Enteritidis (*S.* Enteritidis) [4]. Statistics show the prevalence of *S*. Enteritidis infections to be 43% as compared to 19% of *S*. Typhimurium. These infections have raised the bar of concern for public health. Hence this has led to the identification of multiple virulence factors and pathogenicity islands for determining their role in virulence [5]. *S. enterica* infects host cells with its supramolecular needle-like complex called the Type Three Secretion System (T3SS) [6]. This is encoded by *Salmonella* pathogenicity islands 1 and 2 (SPI-1 and SPI-2) for invasion into epithelial cells and survival within phagocytic cells respectively. Both SPI-1 [7] and SPI-2 [8, 9] are essential factors for *Salmonella* pathogenesis which is mediated through the secretion of effectors via their respective T3SS. The injection of effectors into host cells leads to actin cytoskeletal rearrangements and membrane ruffling, thereby internalizing the bacterium and further mediating pathogen survival and infection persistence [10, 11]. Though SPI-1 and SPI-2 still remain as crucial determinants of virulence; other SPIs and non-SPI genes have also shown significance in *Salmonella* pathogenesis. The deletion of such virulence determinants would aid in analyzing their host interaction or contribution to microbial adaptation [12].

*Salmonella enterica* serovar Enteritidis (*S*. Enteritidis) and Typhimurium (*S*. Typhimurium) cause non-typhoidal self-limiting gastroenteritis with symptoms of fever and diarrhoea in humans [13]. A microarray analysis identified genes essential for colonization and pathogenesis of *S*. Typhimurium both in vitro and in vivo models [14–17]. Another study reported the fast invasion kinetics and SPI-1 dependent inflammation of *S*. Enteritidis in streptomycin pretreated C57BL/6 murine models as compared to SPI-1 deficient *S*. Typhimurium strain [18, 19]. Hence the identification of genes responsible for early colonization of *S*. Enteritidis would be of significant relevance. A study reported that there are certain additional genes present in *S*. Enteritidis for infection establishment as compared to *S*. Typhimurium [20]. This led to the hypothesis of the involvement of these genes in *S*. Enteritidis contributing to greater colonization and host–pathogen interaction. A previous study from our laboratory involving a comparative genome analysis of Enteritidis and Typhimurium serovars showed 98% identity with 2% extra genetic elements likely being involved in the greater infection phenotype of *S*. Enteritidis [18]. Both serovars share similar virulence mechanisms pertaining to epithelial cell invasion and survival within macrophages. In spite of these similarities, both exhibited variations in their infection profiles relating to pathogen persistence in host. It was found that more than 200 differential genes were present in *S*. Enteritidis as compared to Typhimurium most of which were clustered in unique islands gained through horizontal gene transfer termed as “regions of difference” (ROD).

The objective of the present study is to investigate the role of SEN3897, an alanine racemase gene that showed significant differential expression in ROD34 island of *S*. Enteritidis which wasn’t present in *S*. Typhimurium. Alanine racemases (EC 5.1.1.1) are unique pyridoxal-phosphate dependent bacterial enzymes essential for the reversible racemization of l-alanine to d-form [21, 22]. They are ubiquitously present in all prokaryotes and also exceptionally present in eukaryotes like fungi and yeasts to produce d-alanine for peptidoglycan synthesis. Generally either the presence of one or two alanine racemase genes have been reported [23–25]. For example in *Salmonella* Typhimurium; there are two isoforms (non-homologous) of alanine racemase genes (*alr* and *dadX*). The constitutively expressing alanine racemase gene; *alr* is essential for cell wall synthesis by forming d-alanine required for peptidoglycan cross-linking [26]. Another gene *dadX* forms a secondary source for cell-wall biosynthesis though it’s basically required for l-alanine catabolism forming a substrate for d-alanine dehydrogenase (*dadA*). Nonetheless in *S.* Enteritidis; in addition to these two conserved alanine racemase genes, the presence of an additional alanine racemase gene (SEN_*3897*) was quite interesting and speculative in terms of its functional relevance in the pathogen. Moreover bacterial cell wall has always been an interesting target for many antibiotics and antimicrobial agents [27]. Hence this study targets to characterize this uniquely differential alanine racemase gene in *S*. Enteritidis and investigate its role in virulence with a view that it may serve as a potential therapeutic target.

## Methods

### Bacterial strains, plasmids and growth conditions

The bacterial strains and plasmids used in this study are listed in Table 1. The streptomycin resistant wild-type *Salmonella* Enteritidis P125109 and its isogenic mutants were monitored for their growth kinetics by spectrophotometric measurements (OD_600_) in Luria–Bertani (LB) medium (10 g/l tryptone, 5 g/l yeast-extract, 5 g/l NaCl; HiMedia, India) with/without d-alanine supplementation (100 µg/ml) at 37 °C, 150 rpm. The strains were sub-cultured (1:100 ratio) from overnight grown cultures under optimum conditions and absorbance measurements and plating were performed every 1 h till 10 h. The invasion assays required the bacterial strains to be grown overnight at 37 °C 120 rpm in SPI-1 inducing medium (LB containing 0.3 M NaCl) and subsequently subcultured under the similar conditions till its exponential phase. The growth media was supplemented with suitable antibiotics when required: ampicillin (Amp), 100 µg/ml; kanamycin (Km), 50 µg/ml; chloramphenicol (Cm), 20 µg/ml and streptomycin (Sm), 50 µg/ml.

**Table 1:** Bacterial strains and plasmids used in study

	Description	References
Strains		
SENWT	*Salmonella enterica* serovar Enteritidis P125109 wild type; Sm^r^	[18, 79]
SEN∆*alr*	*SENalr* mutant (*SENalr*::*aphT*); Sm^r^ Km^r^	This study
SEN∆*dadX*	*SENdadX* mutant (*SENdadX*::*aphT*); Sm^r^ Km^r^	
SEN∆*3897*	*SEN3897* mutant (*SEN3897::aphT*); Sm^r^ Km^r^	
SEN ∆*alr*∆*dadX*	Double mutant of *SENalr* and *SENdadX* (*SENalr*::*aphT, SENdadX*::*cat*); Sm^r^ Km^r^ Cm^r^	
∆ InvC	*Salmonella* Enteritidis TTSS-1 deficient strain (*SENinvC::aphT*); Sm^r^ Km^r^	[18]
∆ SsaV	*Salmonella* Enteritidis TTSS-2 deficient strain; *ssaV* mutant Sm^r^	[80]
DH5α	*Escherichia coli*	
Codon Plus	*Escherichia coli* (DE3) Codon Plus	
Plasmids		
pKD4	Template plasmid; FRT-*aphT*-FRT (containing kanamycin resistance gene, Km^r^)	[28]
pKD46	Red recombinase expression plasmid (Amp^r^)	[28]
pKD3	Template plasmid carrying chloramphenicol resistance gene flanked by FRT (Cm^r^)	[28]
pCJLA-GFP	GFP-plasmid (Amp^r^)	[81]
pET28a	His tag containing expression vector (Km^r^)	

### Mutant construction

The primers used in this study are listed in Additional file 1: Table S1. The single mutants of alanine racemase genes were generated by one-step inactivation method of the lambda-red recombinase system [28]. This inactivation method required template plasmids namely, pKD4 and pKD3 conferring kanamycin (Km) and chloramphenicol (Cm) resistance respectively. The single mutants were selected on their respective antibiotics and PCR screened with their specific confirmatory primers (Additional file 1: Table S1). The double mutants in *S*. Enteritidis genome were constructed by the conventional phage transduction method. Briefly, the grown culture of the recipient mutant strain (Km^R^) was transduced with the phage lysate of the donor mutant strain (Cm^R^) for 15 min at 37 °C. The transduced colonies were then selected on LB plates with kanamycin (50 µg/ml) supplementation and screened in PCR with FRT-specific primers confirming two antibiotic cassettes within the chromosome (Additional file 1: Table S1). These colonies were subsequently grown in LB supplemented with both chloramphenicol (20 µg/ml) and kanamycin (50 µg/ml) to confirm the integration of both mutations in *S*. Enteritidis genome. In the complemented strain, the *alr* gene expression was controlled under its native promoter. The *alr* gene along with its 1000-bp upstream sequence was cloned in pCH112 plasmid [18] with *Nco*I and *Xba*I restriction enzymes and subsequently transformed in SEN ∆*alr*∆*dadX*.

### Bacterial staining

DAPI (4,6-diamidino-2-phenylindole) and PI (propidium iodide) are nucleic acid stains for differentiation of live cells from damaged/permeabilized ones. DAPI detects all cells uniformly however the nucleic acid intercalation by PI occurs only in impaired bacterial membranes [29, 30]. Hence bacteria with compromised cell membranes are easily stained with PI and they appear red. In this study WT and SEN*∆alr∆dadX* strains were stained with both DAPI and PI to investigate the effect of nutritional limitation (d-alanine: 5, 10, 50 µg/ml) on bacterial cell integrity. The bacterial cells were pelleted at their respective time-points (1 and 10 h). The cells were resuspended and fixed in 4% PFA (paraformaldehyde) for 20 min at room temperature and subsequently centrifuged and washed in PBS. After centrifugation, the bacterial cells were stained initially with DAPI (10 µg/ml) followed by PI (10 µg/ml) with incubation at room temperature for 15 min each in the dark. The excess fluorochromes from cells were removed by pelleting and subsequent washing. 10 μl of each sample was mounted onto the slides with Vectashield mounting medium (Vector Laboratories) and the coverslip was placed for further image acquisition with AMG EVOS Imaging fluorescence microscope (Thermo Fischer) to detect the necessary signals.

### Electron microscopy

The overnight cultures of WT and SEN*∆alr∆dadX* (d-Ala 50 µg/ml) were subcultured to examine the effect of d-alanine supplementation (5, 10, 50 µg/ml) in growth. The subcultured bacterial cells were harvested at specific time-points (1 and 10 h) by centrifugation and washed with PBS to remove residual media contents. These bacterial pellets were fixed in 4% PFA (paraformaldehyde) for 20 min at room temperature and subsequently centrifuged and washed in PBS. The samples were then subjected to dehydration through a gradient of ethanol washings (10–100%) at room temperature. After drying the bacterial specimens were mounted on SEM silicon substrate chips and subsequently observed and analysed in Zeiss EVO 60 scanning electron microscope (Institute of Physics, Bhubaneswar).

### Sequence retrieval and structural modeling

The X-ray crystal structures of alanine racemase proteins from *Salmonella enterica* subsp. *enterica* serovar Enteritidis str. P125109 were unavailable; as a result the protein sequences of locus SEN3897 (protein_id = “WP_000976814.1”), SEN4016 (protein_id = “WP_001147297.1”) and SEN1235 (protein_id = “WP_000197907.1”) were retrieved from NCBI and subjected to I-Tasser for 3D structure modelling [31]. The comparison with homologous structures was also done using BLASTP against PDB database. For global structural similarity, all three proteins were superimposed using matchmaker tool in Chimera [32] in which SEN4016 was taken as the reference structure. The superimposition of proteins provided a sequence alignment panel in which the conserved amino acid residues were presented. The retrieved structures from I-Tasser were subjected to active site analysis using CastP server [33].

### Cloning, protein over-expression and purification

The alanine racemase genes were were PCR amplified from *S*. Enteritidis chromosomal DNA using the designed primers (Additional file 1: Table S1) for cloning in pET28a vector. *Nhe*I and *Xho*I restriction enzymes were used for double digestion of vector and insert and these products were subsequently ligated at 16 °C. The recombinant constructs of pET28a-*alr*, pET28a-*dadX* and pET28a-SEN*3897* were confirmed through DNA sequencing. These recombinant constructs were transformed in *E coli* BL21 (DE3) Codon plus strain for protein expression and selected on kanamycin (50 µg/ml). A pure single colony was grown overnight at 37 °C and was further cultured into fresh 1L LB medium till the exponential phase (A_600_ ~ 0.6). With the desired cell density, the bacterial cells were induced with 1 mM isopropyl-β-d-thiogalactopyranoside (IPTG) and incubated at 18 °C for 12 h. The cells were harvested by centrifugation and resuspended in lysis buffer (50 mM Tris–HCl, pH 8.0, 50 mM NaCl and 10 mM imidazole) containing complete protease inhibitor cocktail tablet (Roche). The cells were sonicated and centrifuged at 13000 rpm for 30 min for cell-free lysate extract. The lysate was loaded onto a column packed with Ni–NTA resin beads (Qiagen) which were already equilibrated with the same buffer. The recombinant alanine racemase proteins carried N-terminal 6X His-tag. After binding, the column was washed with the same buffer and the proteins were eluted with an elution buffer (50 mM Tris–HCl, pH 8.0, 50 mM NaCl) with increasing gradient of imidazole concentration (50, 100, 250 and 500 mM). The eluted fractions of the protein were run on a 12% SDS-PAGE for purity analysis. These fractions were pooled and buffer exchanged for testing the alanine racemase enzymatic activity.

### Enzyme assay

The alanine racemase activity was assayed by a modified spectrophotometric method [34] at 37 °C with l- and d-alanine as substrates. The conversion of d-alanine to l-alanine resulted in the increase of absorbance at 340 nm with the formation of NADH. This mixture comprises of 0.2 U l-alanine dehydrogenase (from *Bacillus subtilis*, Sigma), 10 μM PLP, 20 mM d-alanine and 2.5 mM NAD^+^ in Tris–HCl buffer (pH 8.0). The reaction started with the addition of alanine racemase followed by incubation at 37 °C and the increase in absorbance was monitored with NADH formation.

The assay mixture for the conversion of l-alanine to d-alanine comprised of 20 mM l-alanine, 10 μM PLP, 0.2 mM NADH, 0.5 U d-amino acid aminotransferase (from porcine kidney, Sigma) and 2 U l-lactate dehydrogenase (from *Lactobacillus leichmanii*, Sigma) in Tris–HCl buffer (pH 8.0). The reaction was initiated with alanine racemase addition followed by incubation at 37 °C and the decrease in absorbance at 340 nm was observed with NAD^+^ formation to assess the alanine racemase activity. One unit of enzyme was defined as the amount of substrate (i.e. l- or d-alanine) produced (μmol) per min per mg protein. The absorption spectra was obtained with Cary 60 UV–Vis spectrophotometer (Agilent Technologies).

### Adhesion and invasion assay

Adhesion and invasion assays were performed in human colon cancer epithelial cells (HCT116) as previously described [35, 36]. The cells were grown in 24-well plate format for 14–16 h prior to infection at 1 × 10^5^cells/well density in Dulbecco’s modified Eagle’s medium (DMEM; Gibco) supplemented with 10% fetal bovine serum (FBS) at 37 °C in 5% CO_2_ incubator. d-Alanine (100 µg/ml) was also added to the culture medium under specific conditions. The epithelial cultures were infected with *S*. Enteritidis WT, SEN*∆alr*, SEN*∆dadX*, SEN*∆3897* and SEN*∆alr∆dadX* at an MOI 10 (multiplicity of infection). These bacterial strains were grown overnight and subcultured in SPI-1 inducing medium (0.3 M NaCl) at 120 rpm as mentioned before. The bacterial inoculums and seeded 24-well plate for attachment assay were placed on ice 15 min prior to half an hour infection time-point. In invasion assay after 1 h post-infection the cells were washed and replaced with gentamicin (100 µg/ml) supplemented DMEM with subsequent incubation for 2 h. The cells were then lysed with 0.1% sodium deoxycholate and plated with suitable dilutions for the determination of viable adherent/invasive bacterial counts. These experiments were independently conducted thrice in the form of biological triplicates.

The invasion profile of SEN*∆alr∆dadX* and WT were also determined microscopically before lysing. The cells were fixed with 4% PFA (*v/v*) in PBS for 15–20 min and permeabilized with 0.1% Triton X-100 (*v/v*) with subsequent incubation (20 min) for Alexa546 Phalloidin (Invitrogen) actin staining. The bacterial strains were transformed with pCJLA GFP plasmid for visualization. The coverslips were mounted onto the slides with Vectashield Mounting medium (Vector Laboratories) and the images were acquired with Leica TCSSP5 inverted microscope using a 63× oil immersion objective at Central Instrumentation facility, Institute of Life Sciences, Odisha.

### Growth of alanine racemase mutants in macrophages

The uptake and survival kinetics of alanine racemase mutants was investigated in murine macrophage cell line (RAW264.7) in a 24-well plate set [37, 38]. These cells were cultured in Dulbecco’s modified Eagle’s medium (DMEM) (Gibco) with additional supplementation of 10% Fetal Bovine Serum (FBS) and 1% antibiotics (Penicillin–Streptomycin) and grown for 14–16 h at a density of 1 × 10^5^ cells/well before experimentation. Under certain requirements d-alanine (100 µg/ml) was also added to the culture media. The monolayers were infected with *S*. Enteritidis WT, SEN*∆alr*, SEN*∆dadX*, SEN*∆3897* and SEN*∆alr∆dadX* individually at an MOI 10. One hour post-infection, the monolayers were washed with PBS and replaced with gentamicin supplemented DMEM (100 µg/ml) and incubated at 37 °C, 5% CO_2_. At varied time-points (2 h and 24 h) of post-infection the monolayers were lysed by 0.1% Triton X-100 and plated to determine the bacterial uptake and survival within phagocytic cells at 2 h and 24 h time-points respectively. The bacterial survival within the intact macrophage monolayers was also assessed microscopically at 24 h p.i before lysing (similar to the invasion assay). These assays were done thrice in biological triplicates.

### Quantitative real time PCR assays

RNA was extracted from logarithmic and stationary phase cultures of *S*. Enteritidis by Trizol Reagent (Ambion, USA) to determine the individual expression profile of the alanine racemase genes (*alr*, *dadX* and SEN*3897*) in vitro. RNA extraction was later followed by DNase treatment (Thermo Fischer Scientific, USA) for pure RNA which was used for cDNA synthesis using Revert-aid cDNA Synthesis Kit (Thermo Fischer Scientific, USA). qPCR was performed in triplicates using Kapa Sybr Fast qPCR Master Mix (2×) (Kapa Biosystems, USA) with normalized cDNA templates. The 16s rRNA gene was used as the housekeeping gene.

RNA extraction was also done from *S*. Enteritidis and mutant cultures (SEN*∆alr* and SEN*∆3897*) grown in SPI-1 (0.3 M NaCl LB at 37 °C, 120 rpm) and SPI-2 (minimal media, pH 5.4 with 0.1% casamino acids) inducing medium for investigating their comparative SPI-1 and SPI-2 gene expression profile respectively.

### Mice infection and histopathological evaluation

In this study C57BL/6 specific pathogen free (SPF) mice were bred and maintained at the animal house facility of School of Biotechnology, KIIT University, Odisha for experimentation. The in vivo work followed the guidelines outlined by the Institutional Animal Ethics Committee (IAEC), KIIT University bearing the approval number: KSBT/IAEC/2014/MEET-1/A12. 6–8 week old streptomycin pre-treated mice were used in this study, as previously described [39]. *S*. Enteritidis WT, SEN*∆alr*, SEN*∆dadX*, SEN*∆3897* and SEN*∆alr∆dadX* were grown overnight and sub-cultured (1:20) for 4 h till log phase. The bacterial cultures were harvested and processed individually with equal loads (~ 10^7^ CFU) for oral mice infection (5 in each group). Bacterial loads in feces was determined 24 h and 48 h p.i. Additionally the microbial counts in cecum, mesenteric lymph node (mLN), spleen and liver were assessed at 72 h p.i by plating appropriate dilutions of their respective homogenates on MacConkey agar plates with required antibiotic supplementation.

Histopathological assessment was done as described previously [18, 35]. 5 µm sections of the cryopreserved portions of cecum embedded in OCT were obtained on slides and subsequently stained with hematoxylin and eosin (H&E). The stained sections were evaluated for pathoscore by pathologists on the basis of pathological variation causing sub-mucosal edema, polymorphonuclear neutrophil (PMN) infiltration, goblet cell loss and epithelial ulceration. The range of pathological scores varied from 0 to 13 arbitrary units corresponding to the degree of inflammation. This includes intact intestinal lining displaying no inflammation (pathoscore 0); very mild inflammation (1–2); slight inflammation (3–4); intermediate inflammation (5–8) and significant inflammation (9–13).

### Statistical analysis

All the experiments were conducted independently in biological triplicates to represent the data sets with mean ± standard deviation. One-way analysis of variance (ANOVA) and t-tests were used to examine significant differences during treatments. GraphPad Prism version 6.0 [40] was used for all analyses.

## Results

### Effect of d-alanine starvation on growth and viability

To determine the role of three alanine racemase genes in the survival of *Salmonella* Enteritidis, gene disruptions were carried out using the λ-Red recombination system [28] as mentioned in “Methods” (Additional file 2: Figure S1). The characterization of this unique alanine racemase gene (SEN3897) in *S*. Enteritidis was studied through mutational analysis to investigate its sole functional relevance in the presence or absence of other known alanine racemases. This led to the construction of SEN ∆*alr*∆*dadX* double mutant which required exogenous d-alanine supplementation (50 µg/ml) along with antibiotics in LB agar plates. This double mutant bearing SEN3897 exclusively didn’t show any signs of growth with l-alanine (100 µg/ml) supplementation.

**Fig. 1:**
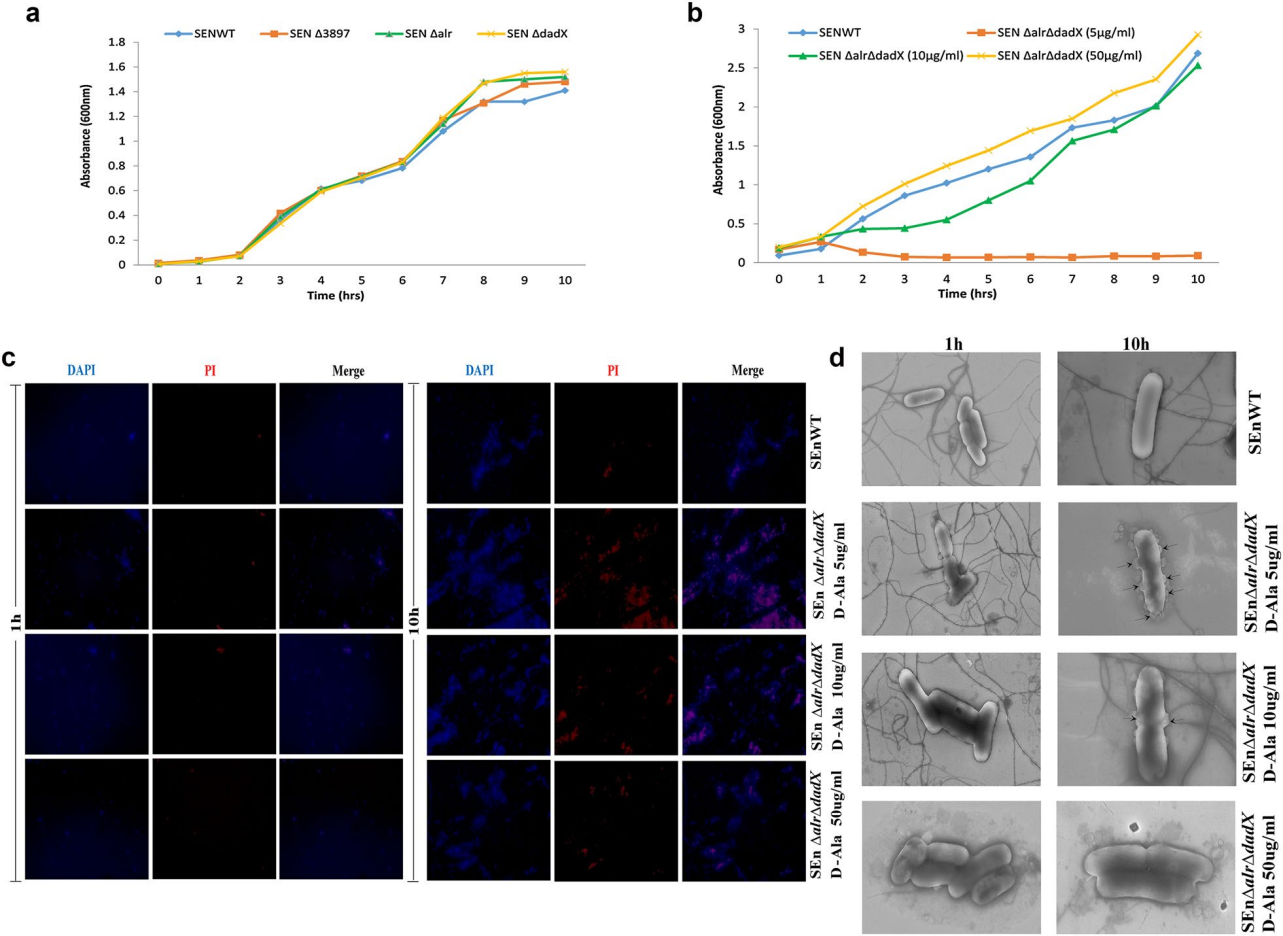
Growth and survival kinetics of the alanine racemase mutants in vitro. **a** Growth curve of SEN *∆alr*, SEN *∆dadX*, SEN *∆3897*, WT *Salmonella* Enteritidis in LB. **b** Growth curve of wild-type *Salmonella* and *alr dadX* double mutant in liquid culture with d-alanine supplementation (5, 10, and 50 µg/ml). Growth was measured by monitoring the OD at 600 nm. **c** Fluorescence microscopy images of d-alanine starvation on cell wall integrity of *alr dadX* double mutant and WT at 1 and 10 h time-points through DAPI/PI dual staining. **d** Scanning electron microscopy images of WT and *alr dadX* double mutant under limited and optimum d-alanine concentration (5, 10 and 50 µg/ml) for observing the morphological changes at 1 h and 10 h time-points. Arrows indicate morphological alterations on cell surface due to d-alanine starvation. Data represent the average of three independent determinations. Scale: 1 µm (1 h time-points), scale: 200 nm (10 h time-points)

The single alanine racemase mutants of SEN*∆alr* (2.4 generations/h), SEN*∆dadX* (2.42 generations/h) and SEN*∆3897* (2.21 generations/h) showed growth rates similar to SENWT (2.46 generations/h) (Fig. 1a; Additional file 3: Figure S2A). However the double mutant strain SEN*∆alr∆dadX* showed growth rates (OD_600_) comparable to WT only when supplied with optimum amount of exogenous d-alanine (50 µg/ml) (Fig. 1b). SEN*∆alr∆dadX* with reduced d-alanine (5 µg/ml) supplementation displayed poor mutant growth (0.53 generations/h). The absorbance at 600 nm (optical density) increased slightly in the first hour then plateaued and consequently decreased till 10 h (Fig. 1b). An increased d-alanine supplementation to 10 µg/ml showed a marginal augmented growth rate of the mutant strain (1.76 generations/h). However with additional d-alanine (50 µg/ml) supplementation the growth rates (2.46 generations/h) were similar to WT (2.49 generations/h) (Fig. 1b) which was corroborated by similar bacterial counts at the respective time-points for WT and mutant strain (50 µg/ml d-ala) (Additional file 3: Figure S2B). These observations indicated that the sole presence of SEN3897 in *Salmonella* Enteritidis may not support growth in the absence of d-alanine supplementation and the depletion of such d-alanine pools results in growth retardation.

### d-Alanine starvation affects membrane integrity

To further investigate the effect of nutritional limitation on reduced viability of the mutant strain SEN*∆alr∆dadX*, we monitored the changes in cell envelope and membrane integrity through DAPI/PI dual staining. The mutant strain SEN*∆alr∆dadX* was grown under three different d-alanine concentrations (5, 10 and 50 µg/ml) to investigate the optimized d-alanine amount for pathogen survival. At 1 h there were no signs of any red signals for either WT or mutant strain (SEN*∆alr∆dadX*). However during starvation (10 h), PI signal intensity increased in the mutant cells (d-Ala 5 µg/ml) possibly due to prolonged nutritional limitation (Fig. 1c) as compared to WT and SEN*∆alr∆dadX* (d-Ala 10 and 50 µg/ml). This is in line with the trend seen in *Lactobacillus plantarum* [41]. The later time-points showed less detection of DAPI signals in mutant cells in the merged panel (Fig. 1c) since prolonged starvation (10 h) allowed PI to enter the mutant cells in massive amounts which interfered with DAPI binding.

The loss of membrane integrity in the mutant strain SEN*∆alr∆dadX* due to prolonged d-alanine starvation was also validated with SEM analysis in a time-dependent kinetic study (10 h) (Fig. 1d). At 1 h time-point, WT and mutant strains grown under d-alanine supplementation (5, 10, and 50 µg/ml) showed similar morphology. At 10 h certain morphological alternations were observed in the mutant strain SEN*∆alr∆dadX* (Fig. 1d). The mutants displayed damaged walls with structural distortions. SEN*∆alr∆dadX* grown under d-alanine supplementation of 10 µg/ml however showed marginal changes in morphology at 10 h. This data correlates with the microscopic observation showing a similar proportion of damaged cells at 10 h (Fig. 1c).

### SEN_3897 shows high fold alanine racemase activity in *S*. Enteritidis

From I-Tasser and BLAST analyses, SEN4016 (359 amino acid residues) showed significant structural identity of 91% with Chain A, Y274f Alanine Racemase from *E. Coli* (PDB-ID-4WR3) with an estimated score of 0.97 ± 0.05 and C-score of 1.82. Similarly, SEN3897 having a score of 0.85 ± 0.08 and C score of 1.00 showed 42% identity with Chain A, Alanine Racemase from *Bartonella henselae* (PDB-ID-3KW3) and SEN1235 with a C score of 1.79 and estimated score of 0.97 ± 0.05 showed 48% identity with chain A of alanine racemase from *Pseudomonas aeruginosa* (PDB-ID-1RCQ). The superimposition of SEN4016, SEN3897 and SEN1235 confirmed their significant structural similarities (Additional file 4: Figure S3B) in the conserved residues from 11 to 60 at N-terminal position and 246–330 at C-terminal. Among the conserved moieties, the N-terminal residues from 41 to 60 and C-terminal residues from 291 to 300 showed significant conservation in all three alanine racemase forms (Additional file 4: Figure S3A). The CastP analysis provided the catalytic active sites of all the three forms of alanine racemase (Additional file 5: Table S2). From STRING analysis, SEN3897 was found to be an isoform of Alr and was termed as Alr3. However Fig. 1 portrayed the inhibition of microbial growth in the sole presence of SEN3897 which raises the possibility of SEN3897 either being dependent on other alanine racemases for d-alanine synthesis or for not encoding a functional alanine racemase.

**Fig. 2:**
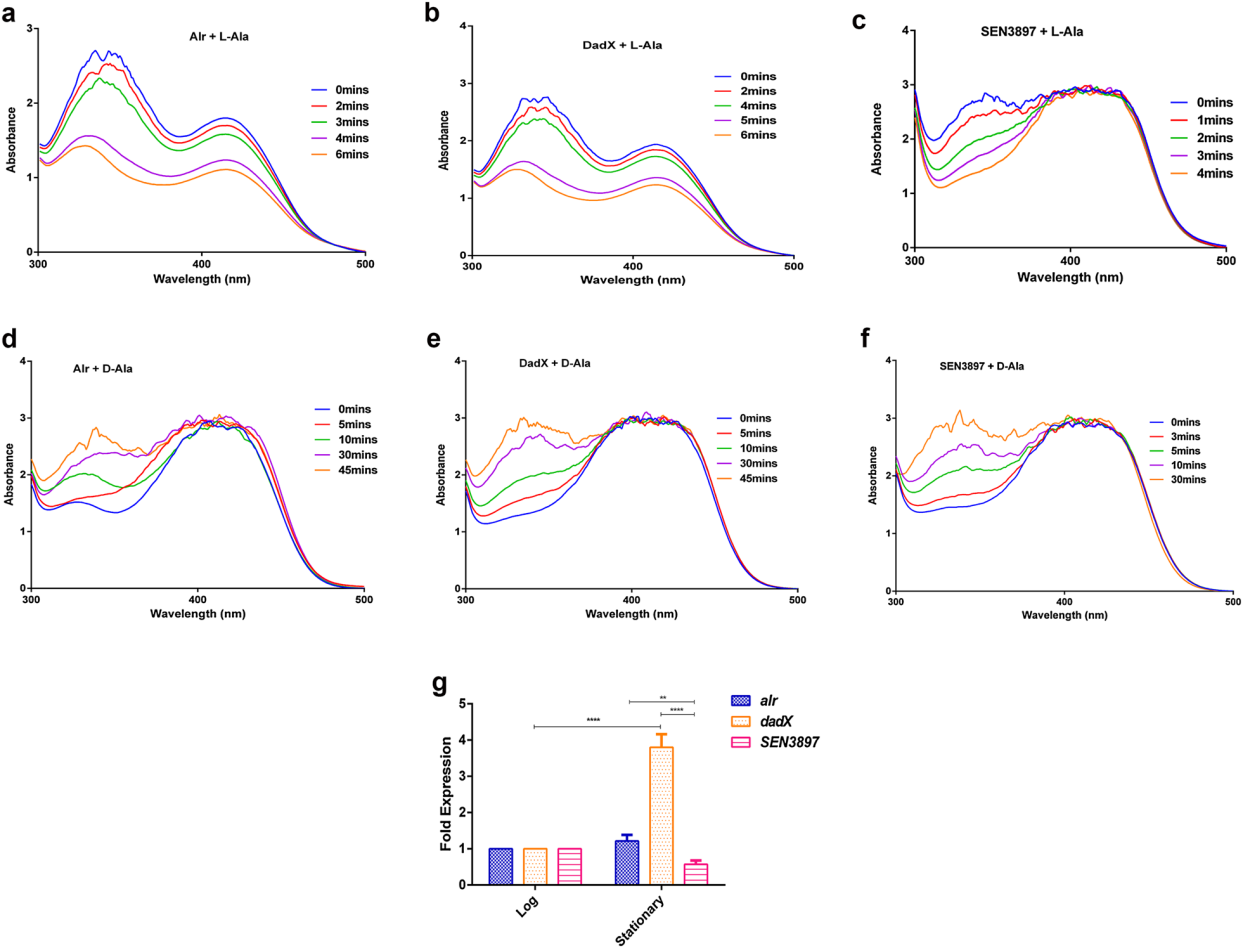
Spectral change of Alr, DadX and SEN3897 alanine racemases caused by incubation with (**a**–**c**) l-alanine and (**d**–**f**) d-alanine. The UV–visible spectra (300–500 nm) was obtained at the indicated times. **g** Expression of *alr*, *dadX* and *SEN3897* in vitro during their exponential and stationary growth phases through qRT-PCR analysis

To further explore this hypothesis, we investigated the in vitro alanine racemase activity of SEN3897 by a d-amino acid oxidase coupled assay at 37 °C for l-alanine to d-alanine conversion with PLP. With l-alanine as a substrate the specific activity of SEN3897 (297 µmol/(min mg)) was higher as compared to Alr (213 µmol/(min mg)) and DadX (207 µmol/(min mg)) (Fig. 2a–c). Moreover when d-alanine was used as a substrate, SEN3897 showed a slight increased specific activity (109.3 µmol/(min mg)) relative to Alr (68.4 µmol/(min mg)) and DadX (70.8 µmol/(min mg)) (Fig. 2d–f). The alanine racemase enzyme activity followed Michaelis–Menten kinetics in a varied range of substrate concentrations. With l-alanine as the substrate, the substrate affinity constant (K_m_) of Alr and DadX was 62.1 ± 4 and 58.9 ± 2.7 mM respectively (Table 2). However d-alanine as substrate showed K_m_ values for Alr and DadX as 66.6 ± 4.3 and 52.8 ± 3 mM respectively. The reaction rate of SEN3897 with l-ala and d-ala substrate concentration had a calculated K_m_ value of 89 ± 10.2 and 35 ± 6 mM respectively (Table 2). Accordingly the maximal velocity (V_max_) differed for the enzymes with different enantiomeric forms (Table 2). The K_m_ value of Alr is mostly similar for l- and d-alanine substrates which denotes similar affinity of the enzyme for both the enantiomers. Nevertheless Alr showed a greater binding capacity for l-alanine with less V_max_ value (276.5 ± 5.6 µmol/(min mg)) as compared to SEN3897 (400 ± 25.6 µmol/(min mg)).

**Table 2:** Kinetic parameters of *Salmonella* alanine racemases

	l → d		d → l	
	K_m_ (mM)	V_max_ (µmol/(min mg))	K_m_ (mM)	V_max_ (µmol/(min mg))
Alr	62.1 ± 4	276.5 ± 5.6	66.6 ± 4.3	157 ± 5.3
DadX	58.9 ± 2.7	258 ± 4.9	52.8 ± 3	147 ± 7.4
SEN3897	89.15 ± 10.2	400 ± 25.6	35 ± 6	132.5 ± 11.3

Although SEN3897 showed suitable racemase activity, it wasn’t able to rescue d-alanine deficiency by itself in *alr dadX* double mutant. To elucidate the d-alanine dependency of SEN*3897*, we investigated the expression levels of *alr*, *dadX* and SEN*3897* in LB growth media. The expression of SEN*3897* was two- and fourfold down-regulated relative to *alr* and *dadX* respectively (Fig. 2g). Moreover with the absence of *alr* and *dadX* genes, the 1.5-fold reduced expression of SEN*3897* in SEN *∆alr∆dadX* relative to WT was possibly insufficient to compensate for d-alanine scarcity in the auxotroph (Additional file 6: Figure S4). Therefore the racemase activity of SEN3897 could not completely support d-alanine prototrophy due to its inadequate expression level. Thus, this the lower abundance of SEN*3897* possibly led to d-alanine dependency in the absence of both *alr* and *dadX* in SEN *∆alr∆dadX*.

### SEN*∆3897* and d-alanine auxotrophic mutant of *S*. Enteritidis displayed reduced invasion and survival phenotype in vitro

**Fig. 3:**
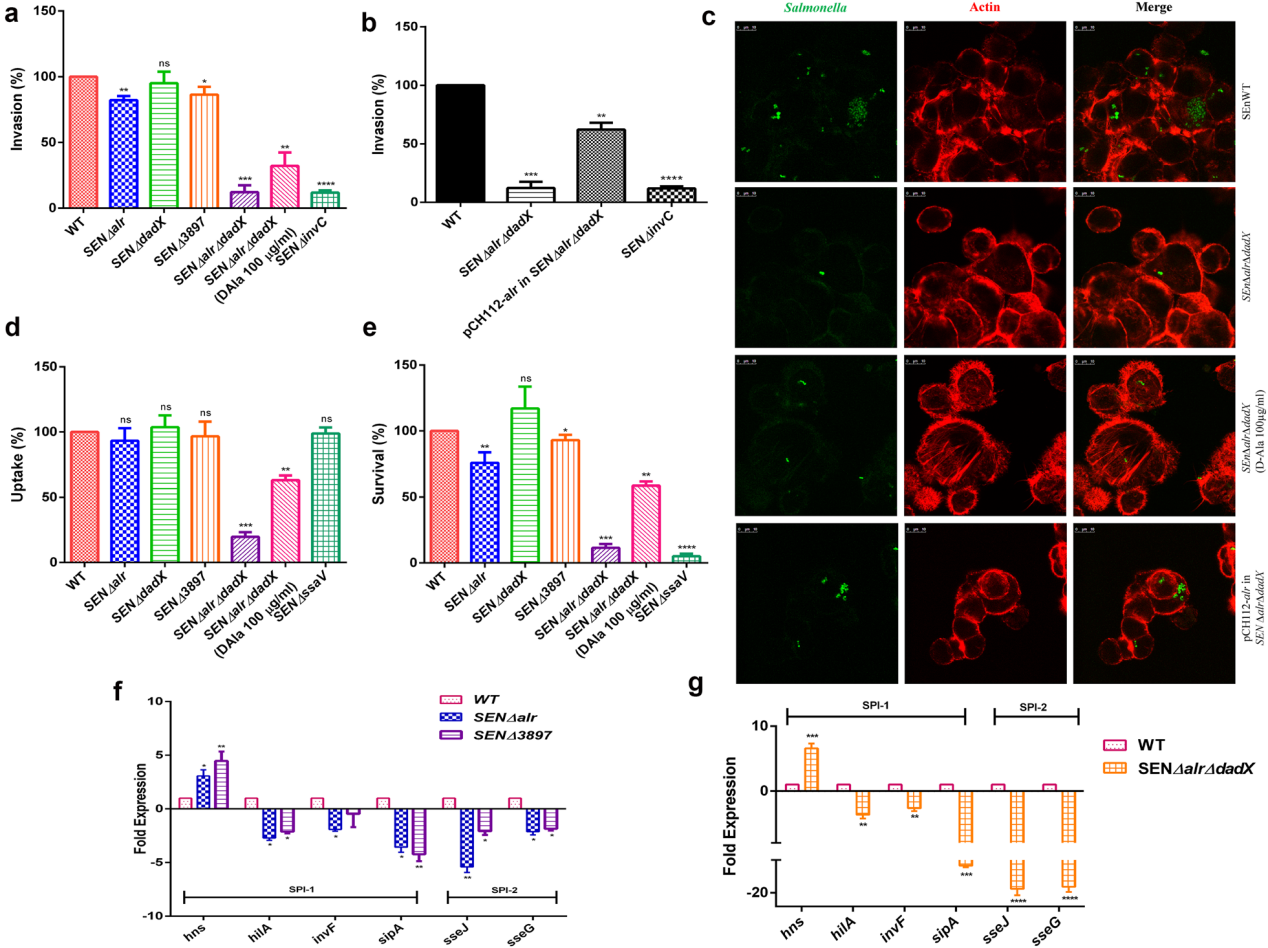
In vitro characterization of alanine racemase mutants. **a**, **b** Invasion assay of SEN *∆alr*, SEN *∆dadX*, SEN *∆3897*, SEN *∆alr∆dadX*, Alr complemented strain (pCH112-*alr* in SEN *∆alr∆dadX*) and WT in HCT116 cell-lines. d-Alanine (100 µg/ml) was added in cell culture media 1 h before infection. **c** Confocal microscopy images of HCT116 cells infected with WT and SEN *∆alr∆dadX* for 50 min at MOI 100, gentamicin treated for 2 h and fixed. Cells were stained for actin with Alexa Fluor 546 Phalloidin (Red) for 15 min at room temperature. *Salmonella* strains were GFP-tagged shown in green and actin in red. Scale bar: 10 μm. **d** Uptake Assay and **e** Survival Assay of SEN *∆alr*, SEN *∆dadX*, SEN *∆3897*, SEN *∆alr∆dadX* and WT in RAW264.7 in murine macrophages at 2 and 24 h time-points respectively. d-Alanine (100 µg/ml) was added in cell culture media 1 h before infection **f** SPI-1/SPI-2 gene expression of WT, SEN *∆alr* and SEN *∆3897* through qRT-PCR analysis. All experiments were performed in triplicate with data represented as mean ± SD. *RT* room temperature. Statistical significance: *P < 0.05, **P < 0.01, ***P < 0.001, ****P < 0.0001

*Salmonella* pathogenesis begins through adhesion into epithelial cells followed by invasion. In vitro characterization of the three alanine racemase genes was carried out in HCT116 cells (colon carcinoma cells) for investigating their role in adhesion and invasion in vitro. The HCT116 cells were infected with WT, SEN *∆alr*, SEN *∆dadX*, SEN *∆3897* and SEN *∆alr∆dadX* with an MOI 10. To aid in comparison we normalized all bacterial counts with WT (100%). SEN *∆alr*, SEN *∆dadX* and SEN *∆3897* strains displayed adhesion percentages of 110, 105 and 115% respectively as compared to WT (Additional file 7: Figure S5). SEN *∆alr* and SEN *∆3897* mutants exhibited significant reduced invasion efficiencies of approximately 80 and 86% in vitro respectively (Fig. 3a). SEN *∆dadX* displayed similar invasion profile (95%) relative to WT. SEN *∆alr∆dadX* displayed a significant lower invasion percentage of 11.1% relative to WT (Fig. 3a). The invC mutant is an invasion deficient strain used as an experimental control. To further reinforce the role of SEN3897, *alr* was cloned in pCH112 plasmid with its native promoter in SEN *∆alr∆dadX* mutant. Interestingly, the growth phenotype was restored in the complemented strain without d-alanine supplementation. Alr complementation also led to the restoration of the in vitro phenotype that wasn’t exclusively maintained by SEN3897 (Fig. 3b). The phenotype restoration due to Alr complementation was also validated through microscopy with increased GFP expression of complemented strain relative to SEN *∆alr∆dadX* mutant (Fig. 3c). The pre-inoculum densities (PID) of SEN*∆alr*, SEN*∆3897*, SEN*∆dadX* and SEN *∆alr∆dadX* prior to infection were 2.0 * 10^6^, 1.7 * 10^6^, 1.9 * 10^6^ and 1.3 * 10^6^ respectively relative to WT (1.5 * 10^6^).

The alanine racemase mutants were assessed for their intracellular survival inside macrophages. The murine macrophage cells, i.e. RAW264.7 were infected with the single mutants of alanine racemase genes at MOI 10. The *alr* and SEN*3897* single mutants showed approximately 93 and 96% uptake in RAW264.7 cells while SEN *∆dadX* displayed approximately 102% uptake as compared to WT (Fig. 3d). However SEN *∆alr∆dadX* strain (d-Ala auxotroph) showed a substantial lower uptake percentage of 21.3% as compared to WT (Fig. 3d). With regards to intracellular survival within macrophages, SEN *∆alr* displayed an attenuated phenotype with 70% CFU as compared to WT (Fig. 3e). Similarly SEN*3897* mutants showed marginal diminished survival with almost 90% surviving pathogen counts in phagocytic cells. Interestingly, SEN *∆alr∆dadX* (12.7%) displayed significantly impaired survival within macrophages (Fig. 3e). SEN *∆ssaV* is an experimental negative control for macrophage survival. The PID of SEN*∆alr*, SEN*∆3897*, SEN*∆dadX* and SEN *∆alr∆dadX* before macrophage assays were 2.5 * 10^6^, 3.1 * 10^6^, 2.8 * 10^6^ and 2.2 * 10^6^ respectively relative to WT (2.6 * 10^6^). Alr complementation in SEN *∆alr∆dadX* restored the survival phenotype (55%) of the d-alanine auxotrophic strain in phagocytic cells (Additional file 8: Figure S6). The microscopy images for intracellular survival of the single alanine racemase mutants (SEN *∆alr,* SEN *∆dadX*, SEN *∆3897*) with GFP expressing plasmid confirmed their respective replication fold phenotype in vitro (Additional file 9: Figure S7).

### d-Alanine supplementation restores the virulence phenotype in vitro

As the *alr dadX* double mutant was shown to require exogenous D-Ala supplementation for growth, we hypothesized that d-alanine limitation might affect its virulence in host. Due to lack of d-amino acids in eukaryotic cells, it’s expected that a d-alanine auxotrophic strain (SEN *∆alr∆dadX*) would have growth defects due to its inability to synthesize d-alanine. This in turn might attenuate the replicative ability of the pathogen in the host cell cytosol. As expected, SEN *∆alr∆dadX* displayed significantly reduced pathogenic loads of 11.1% in epithelial cells as compared to WT (Fig. 3a); though both had equal inoculum densities. We subsequently supplemented the tissue culture medium with d-Ala (100 µg/ml) before being infected with SEN *∆alr∆dadX* to examine their phenotypic changes. It was observed that d-Ala supplementation increased the invasion profile of SEN *∆alr∆dadX* in epithelial cells to 30.1% (Fig. 3a–c). Similarly SEN *∆alr∆dadX* showed impaired uptake percentage of 21.3% and attenuated replication profile of 12.7% in murine macrophages (Fig. 3d, e). d-Alanine supplementation to culture media of RAW264.7 cells therefore increased the uptake percentage for SEN *∆alr∆dadX* to 58.6% at 100 µg/ml d-alanine concentration (Fig. 3d). The intracellular replication fold of SEN *∆alr∆dadX* displayed almost similar augmented profiles as uptake ratios with increased d-Ala supplementation (Fig. 3e). This result is in line with the study linking d-ala inadequacy to decreased pathogenic loads in the nutrient-limiting macrophage environment [42, 43].

Similar assays were also conducted by supplementing the tissue culture medium with d-Ala (50 µg/ml) before infection. It was observed that SEN *∆alr∆dadX* showed reduced restoration of virulence phenotype in both HCT116 and macrophages (Data not shown). SEN *∆alr∆dadX* required an optimum d-alanine (50 µg/ml) quantity for bacterial growth; however with greater concentrations of d-alanine supplementation in culture media, the virulence profile changes. Hence 100 µg/ml d-alanine supplementation was considered over 50 µg/ml d-alanine amounts in culture media for virulence restoration.

### Deletion of *alr* and SEN*3897* affects *Salmonella* virulence in vitro

The inactivation of *alr and* SEN3897 resulted in reduced pathogen invasion and survival in non-phagocytic and phagocytic cells respectively. To this effect the expression profile of SPI-1 effectors and regulators were determined by q-PCR to investigate the reduced invasion phenotype of SEN*∆alr* and SEN*∆3897*. *hilA* transcripts in SEN*∆alr* and SEN*∆3897* showed significant 2.5-fold down-regulation and 2-fold down-regulation respectively relative to WT under SPI-1 conditions (Fig. 3f). *hns* levels in SEN*∆alr* and SEN*∆3897* increased to three- and fourfold respectively when compared to WT (Fig. 3f). The *invF* expression in SEN*∆alr* was observed to be twofold down-regulated thereby resulting in likely reduced *sipA* effector secretion (threefold less). Epithelial cell entry requires active SPI-1 effectors among which *sipA* plays a vital role. In SEN *∆3897*, *sipA* displayed a significant fourfold reduction in expression as compared to WT (Fig. 3f). SEN*∆alr* and SEN*∆3897* also displayed a replication-defective phenotype in macrophages and SPI-2 genes are known to play a major role in this phenotype (Fig. 3e). Hence SPI-2 effectors were assessed through qPCR for SEN*∆alr* and SEN*∆3897.* SEN *∆alr* exhibited attenuated expression profiles of SPI-2 effector genes *sseJ* and *sseG* (~fivefold and ~threefold down-regulation respectively) relative to WT (Fig. 3f). Similarly SEN*∆3897* displayed approximately twofold reduced expression of both *sseJ* and *sseG* as compared to WT. 16s rRNA gene was used as the internal control.

Moreover the deletion of both the essential alanine racemase genes (*alr* and *dadX*) in the d-alanine auxotroph also showed a significant effect on *Salmonella* invasion and survival in epithelial cells and macrophages respectively (Fig. 3a–e). To address this, the mRNA transcripts of SPI-1 and SPI-2 genes (apparatus genes, effectors and regulators) in SEN *∆alr∆dadX* were determined relative to WT to comprehend its phenotype. *hilA* and *hns* levels in SEN *∆alr∆dadX* showed significant 3.5-fold down-regulation and 6-fold up-regulation respectively relative to WT (Fig. 3g). Similarly *invF* levels were 2.5-fold down-regulated which possibly induced less *sipA* production (10-fold less). SEN *∆alr∆dadX* showed attenuated survival within macrophages; hence SPI-2 genes were also examined through qPCR in SEN *∆alr∆dadX* as they are responsible for pathogen survival within macrophages. SEN *∆alr∆dadX* exhibited extremely low transcripts of SPI-2 encoded effector genes like *sseJ* and *sseG* (~18-fold decreased expression) as compared to WT (Fig. 3g). This indicated that the deletion of essential alanine racemases in d-alanine auxotroph possibly modulated the expression of key genes related to *Salmonellosis*.

### In vivo attenuation of SEN*∆3897* and *alr dadX* double mutant

**Fig. 4:**
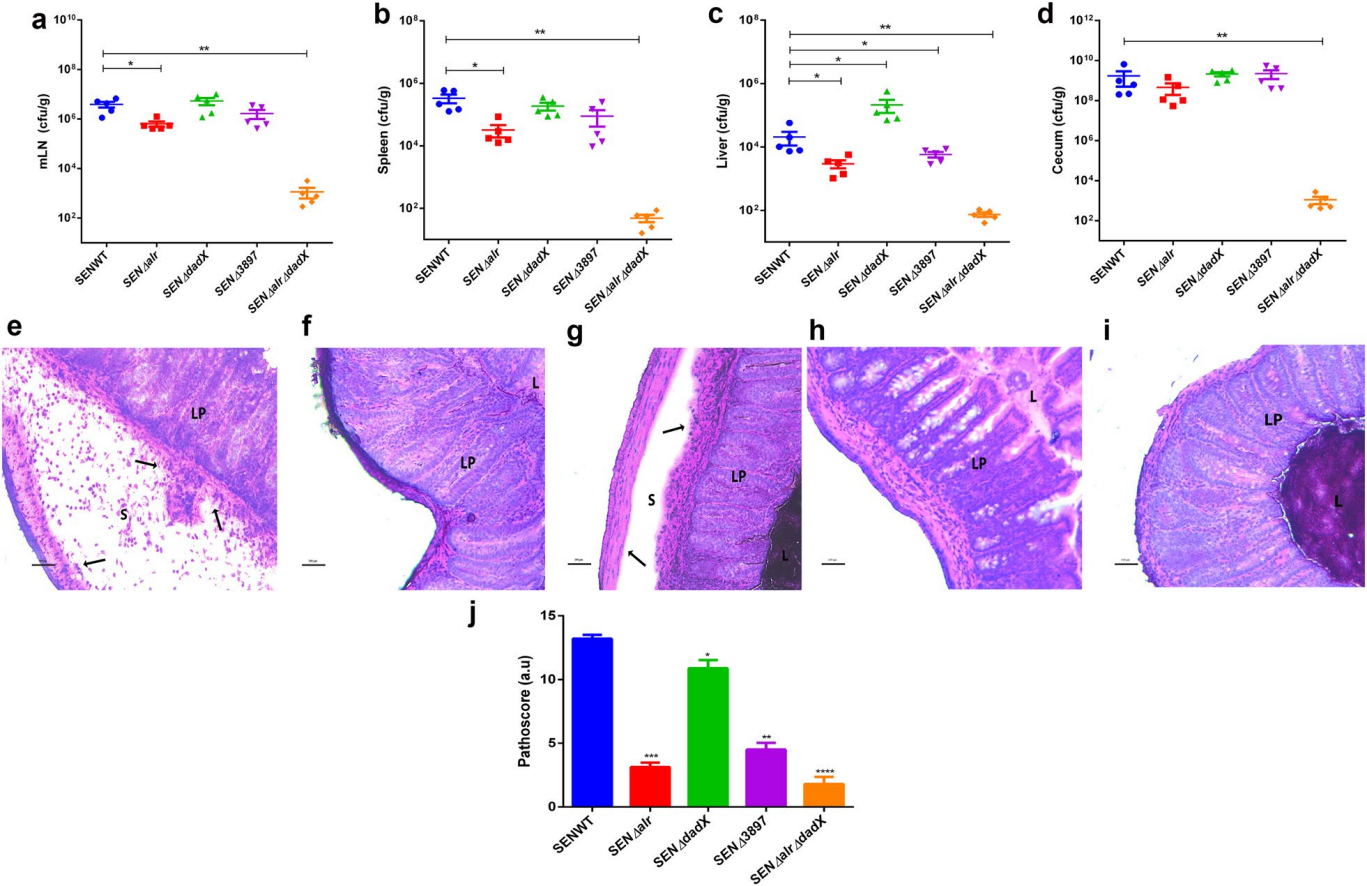
Colonization and cecal inflammation by WT, SEN *∆alr*, SEN *∆dadX*, SEN *∆3897* and SEN *∆alr∆dadX* of *S*. Enteritidis in C57BL/6 mice. **a**–**d** Streptomycin-pretreated mice were infected with the above strains, sacrificed 72 h p.i. and bacterial loads in mesenteric lymph node (mLN), spleen, liver and cecum were determined. **e**–**i** Histopathological assessment of the cecal tissue from animals infected with WT, SEN *∆alr*, SEN *∆dadX* and SEN *∆*3897 and SEN *∆alr∆dadX*. **j** Cecal pathological analysis by semiquantitative method. Bars, 100 µm. *S* submucosa, *L* lumen, *LP* lamina propria. Statistical significance: *P < 0.05, **P < 0.01, ***P < 0.001, ****P < 0.0001

The in vivo characterization of alanine racemase mutants was performed in C57BL/6 mice. Streptomycin pretreated mice (n = 5) were infected with ~10^7^ CFU of alanine racemase mutant strains (SEN *∆alr*, SEN *∆dadX*, SEN *∆3897* and SEN *∆alr∆dadX*) and WT. The colonization levels were comparable between WT and the mutants at 24 h post infection (Additional file 10: Figure S8). However 1.0-log_10_ fold decreased colonization was observed in SEN *∆alr* at 48 h post infection relative to WT (Additional file 10: Figure S8). The mesenteric lymph node (mLN) displayed equivalent bacterial counts of the single mutants except SEN *∆alr* which showed slightly reduced count relative to the WT at 72 h post infection (Fig. 4a). SEN *∆alr* also exhibited 1.0-log_10_ fold reduced ability to replicate and survive in splenic sites at 72 h post infection (Fig. 4b). In the liver, SEN *∆alr* and SEN *∆3897* portrayed 0.8-log_10_ fold and 0.5-log_10_ fold reduced systemic loads as compared to WT at the same timepoint (Fig. 4c). All single mutants showed equivalent pathogenic loads in cecum relative to WT at 72 h post infection (Fig. 4d). SENWT depicted a pronounced intestinal inflammation (Fig. 4e) at 72 h p.i. SEN *∆alr* showed mild to no inflammation phenotype in vivo (Fig. 4f) during the similar time interval. Conversely, the SEN *∆dadX* mutant strain (Fig. 4g) showed inflammation profile similar to WT at 72 h p.i. SEN *∆3897* displayed signs of intermediate cecal inflammation at 72 h post infection (Fig. 4h). Conversely, the SEN *∆dadX* mutant strain (Fig. 4g) had a pronounced intestinal inflammation relative to WT (Fig. 4e) at 72 h p.i. The attenuation in inflammation of SEN *∆alr* and SEN *∆3897* presented respective cecal pathoscores as 3.5 and 4.5 while the cecal sections infected with WT and SEN *∆dadX* had pathoscores of 13.3 and 9.7 respectively (Fig. 4j).

On the contrary SEN *∆alr∆dadX* portrayed significantly reduced colonization efficiency at 24 h p.i. (3-log_10_ fold less) and 48 h p.i. (4-log_10_ fold less) in comparison to WT (Additional file 10: Figure S8). The less colonized numbers of SEN *∆alr∆dadX* strain showed 3-log_10_ fold reduction in bacterial counts in mLN, spleen and liver at 72 h p.i (Fig. 4a–c). A significant loss in colonization (6-log_10_ fold less) was also observed in cecum at 72 h post infection (Fig. 4d). SEN *∆alr∆dadX* strain was highly attenuated and the cecal mucosa displayed little to no inflammation (Fig. 4i) at 72 h post infection with a pathoscore of 2.1 (Fig. 4j) as compared to WT (pathoscore of 13.3). These observations reflect the scarcity of d-alanine in the double mutant that possibly interferes with the establishment of infection in mice. Hence it may be concluded that the presence of SEN*3897* alone cannot support the viability of *Salmonella* in vivo.

## Discussion

Due to the development of drug-resistant enteric pathogens, novel drug design requires improved formulations and strategies. Bacterial cell walls have always been a fascinating target for the development of new antimicrobials [44]. Alanine racemase is an essential prokaryotic homodimeric PLP dependent enzyme catalyzing the reversible racemization of l- to d-alanine. d-alanine forms an integral component for peptidoglycan synthesis and is therefore essential for bacterial growth and survival. *Salmonella* Typhimurium comprises of two isoforms of alanine racemase genes namely *alr* and *dadX* [26]. The constitutively expressing Alr has the primary biosynthetic function of cell wall synthesis and DadX acts as the catabolic alanine racemase which can secondarily act for peptidoglycan cross-linking [45, 46]. Genomic comparison between *S*. Enteritidis and *S*. Typhimurium led to the identification of a third alanine racemase in *S*. Enteritidis. The presence of a third racemase gene was quite intriguing as the presence of either one or two genes has been reported in every microbe [23, 24]. This study has discussed the characterization and contribution of three alanine racemase genes of *Salmonella* Enteritidis with a focus on this newly identified racemase gene SEN3897. This is the first study to report the presence of a third alanine racemase gene in *Salmonella* Enteritidis. The functional role of this unique alanine racemase gene (SEN*3897*) in *S*. Enteritidis was studied through its sole chromosomal inactivation and presence irrespective of other known alanine racemases to investigate its distinguished role in the pathogen.

Among the mutant strains (SEN *∆alr*, SEN *∆dadX*, SEN *∆3897*), the double mutant *SEN*∆*alr*∆*dadX* (sole presence of SEN3897) displayed signs of growth defects, membrane integrity loss and structural modifications with the depletion of d-alanine pools. This indicated that the presence of SEN3897 only couldn’t entirely contribute to d-alanine prototrophy. One similar study had also investigated cellular damage and enhanced permeability due to d-alanine auxotrophy in *A. hydrophila* alanine racemase mutant [47]. Depletion of intracellular d-alanine amounts had similarly resulted in cell death with decreased optical density in many Gram-positive and Gram-negative bacteria like *E. coli, Bacillus subtilis* and *Lactobacillus plantarum* [22, 41, 43, 48–51]. DadX forms a secondary source for d-alanine production in the absence of Alr. Nevertheless SEN_3897 seemingly fails in d-alanine production in the absence of both Alr and DadX. This shows that the *alr* gene when inactivated probably requires either another copy/isoform for survival or d-alanine supplementation in the growth medium. Some studies have reported a single deletion of *alr* gene to be responsible for d-alanine auxotrophy in *Lactobacillus plantarum* or a double knockout of two essential alanine racemases to show d-alanine dependency in *Salmonella Typhimurium* [41, 52]. However our study showed the contribution of a unique alanine racemase gene in strict d-alanine dependency which differs from other related findings.

Alanine racemase genes have been well identified, cloned and expressed in varied microorganisms [24, 26, 45]. Our results presented that the specific activity of SEN3897 was higher than Alr and DadX. The results also indicated SEN3897 to be less reactive with l-alanine relative to d-alanine. Alanine racemases undergo reaction catalysis with a “two-base” mechanism by utilizing two active site residues, i.e., lysine and tyrosine for substrate recognition and conversion [53–55]. The active site of Alr in *S.* Enteritidis is formed by two catalytic residues Lys34 and Tyr343 from two monomers [45, 56] which are conserved among different Alrs in microbes [57, 58]. Similarly SEN3897 contains Lys42 and Tyr349 catalytic active site residues whereas DadX contains only Tyr 449 (Additional file 5: Table S2).

Alanine racemases are ubiquitously present in all prokaryotes and their deletion has been reported to be lethal for pathogen survival in the absence of d-alanine supplementation [22, 41]. Impairment of intracellular survival in macrophages has also been witnessed in alanine racemase mutants or during nutrient deprivation in phagocytes [42]. Our in vitro survival assays in macrophages displayed the role of biosynthetic alanine racemase gene (*alr*) in survival rather than the catabolic gene (*dadX*). d-Alanine catabolism for ATP generation could possibly be carried out by other alternative metabolic pathways in the DadX mutant. However Alr is majorly involved in cell wall biosynthesis; therefore deletion of Alr was expected to cause disruption in cell wall formation thereby affecting growth and viability. Alr mutants of *M. smegmatis* have d-alanine dependency [49]. Additionally these mutants of *M. smegmatis* have 80% reduced survival in macrophages [43]. The inactivation of essential metabolic genes in SEN *∆alr∆dadX* led to diminished intracellular survival of pathogens in our study. This is mostly due to nutrient limitation in the phagosomal compartment as compared to the rich cytosol [59–62] which couldn’t be restored even with the presence of SEN*3897* in SEN*∆alr∆dadX*. Furthermore d-alanine deficiency affected *Salmonella*’s entry into the rich host cytosolic compartment in SEN *∆alr∆dadX* that was subsequently restored with d-Ala addition to cell culture media. Hence, this study showed that the sole presence of SEN3897 was responsible for d-alanine auxotrophy, morphological alterations and reduced pathogen survival. Further a double mutant of *alr* and SEN*3897* was also investigated for the additive effects of mutation in contributing to invasion and survival within epithelial cells and macrophages respectively in vitro. Interestingly, SEN*∆alr∆3897* showed comparable phenotypes with WT in terms of epithelial cell invasion (Additional file 11: Figure S9A) and macrophage uptake at 2 h post infection (Additional file 11: Figure S9B). However this double mutant displayed approximately 1.5-fold increased survival within macrophages at 24 h p.i. relative to WT (Additional file 11: Figure S9C). Since both SEN*∆alr* and SEN*∆3897* single mutants showed attenuation within epithelial cells and macrophages, a double mutant of these two genes may be expected to show an attenuated profile within the same cell lines in vitro. However the variation in the phenotypes between the individual mutants and their double mutant possibly suggests the involvement of complex multi-pathway interactions that displayed synergistic effect of mutations affecting the bacterial system for adapting to its host environment. Additionally the presence or absence of *dadX* by itself didn’t affect colonization levels in SEN*∆alr∆3897* and SEN*∆dadX* respectively indicating that DadX (catabolic alanine racemase) may not be crucial for *Salmonella* pathogenesis. Comparatively the presence of either *alr* or SEN*3897* with *dadX* in SEN*∆3897* and SEN*∆alr* respectively showed reduced colitis which indicates the essential involvement of SEN*alr* and SEN*3897* in virulence.

In *Salmonella*, SPIs are known to play a pivotal role in epithelial cell invasion and intracellular survival [7, 63, 64]. Hence we investigated if the mutants of *alr* and SEN*3897* could possibly affect SPI-1 and SPI-2 through this regulatory pathway for its invasion-defective and attenuated survival phenotype respectively. H-NS is a global repressor of SPI-1 regulators (HilA, HilD, HilC, RtsA) [65] as well as SPI-2 machinery (*ssaB*, *ssaG*). H-NS silences *hilA* expression which affects the invasion genes [66]. HilA plays an essential role in invasion since its inactivation affects the entire SPI-1 locus [67, 68]. This transcriptional activator induces InvF expression and together they coordinate regulated control over SPI-1 effectors [69]. The invasion genes are also regulated by HilD which activates HilA in vitro [70]. We observed a down-regulation of these essential SPI-1 regulators and effectors in SEN *∆alr* and SEN*∆3897*. Similar to SPI-1, we observed significant down-regulation of crucial SPI-2 effectors (*sseJ*, *sseG*) in SEN *∆alr* and SEN*∆3897*. Effectors like SseJ and SseG contribute to successful *Salmonella* replication by inducing SIF formation and stabilizing SCV membrane integrity [66, 71]. SseJ is identical to acyltransferase/lipase which localizes during infection to affect SIF formation [72, 73]. SseF and SseG share sequence similarity and are associated with SCV migration in proximity to Golgi apparatus [74]. Hence our work suggests a combined effect of nutrient deprivation and SPI modulation by SEN3897 to affect pathogen survival in vitro. We also found increased expression of *hns* contributing to reduced levels of *hilA* and other downstream effectors in *SEN*∆*alr*∆*dadX* which explained the reduced phenotype of epithelial cell invasion in the *alr dadX* double mutant. Additionally, in this double mutant, the SPI-2 genes (*sseJ*, *sseG*) also showed immense down-regulation compared to the transcript levels of these crucial effectors observed in *SEN*∆*alr* and SEN∆*3897.*

Our in vivo studies portrayed attenuation in gut inflammation of SEN*∆3897* at 72 h p.i. relative to WT. The attenuation in intestinal inflammation could be possibly due to the reduced replication and survival of SEN*∆3897* in murine macrophages. However there wasn’t any sort of attenuation observed in cecal colonization possibly due to the differences in host system. It has been reported that SPI-1 is likely to be responsible for attenuation in gut inflammation and inflammatory responses via the needle complex, T3SS-1 [75]. This study showed decreased expression of the crucial SPI-1 effectors in SEN *∆3897* relative to WT which likely resulted in reduced inflammatory responses in vivo. SPI-2 is also essential modulator of host inflammatory responses [75]. SEN *∆3897* also showed a down-regulation in the expression of SPI-2 effectors (*sseJ*, *sseG*). Hence investigation into its coordinated regulation by different pathogenicity islands and regulators will help us develop novel insights that may be exploited for development of antibacterial therapy against *Salmonella*. This study portrays a complex network of regulators and virulence-associated factors governing pathogen’s invasion and survival strategies within host [66]. In vivo, the *alr dadX* double mutant displayed hyperattenuation in which the d-alanine synthesis route was possibly inactivated that led to its defective survival and colonization at 72 h p.i. Moreover if d-Ala was present in murine macrophages and mice models; their levels are much below threshold to support growth of the d-alanine auxotrophic strain which lacked the essential alanine racemase genes *alr* and *dadX* for infection establishment. Hence the major finding of this study stated that the sole presence of the third alanine racemase gene; SEN*3897* couldn’t support pathogen survival in vivo and led to strict d-alanine auxotrophy. Moreover deletion of this gene led to defective SPI-1 and SPI-2 functioning; hence this work helped in understanding the link between metabolic genes and their role in *Salmonell*a virulence. Cell wall auxotrophy was also studied for reduced pathogenesis in *Shigella* [76].

The first drug for alanine racemase was cycloserine [24, 77]. Even though Alanine racemase is a key target for antibacterial drug design, the inhibitors of alanine racemase do not have clinical utility because of their lack of specific targets which promotes activity against other PLP-dependent proteins. The Alr inhibitor DCS (d-cycloserine) had major health implications on human nervous system which necessitated the requirement for novel inhibitors with better specificity and less host toxicity. Subsequently the inhibitor development through structure-based studies would examine the dimer interfaces and active site pockets of alanine racemases for use as potential targets against microbial infections. The inhibitor-protein interaction should be aimed for the discovery of new antibiotics with high selectively and specificity for alanine racemase. Another study examined a d-alanine auxotroph as a live vaccine candidate against *Staphylococcus aureus* infection [78]. Our study also examined the role of a d-alanine auxotrophic strain in *Salmonella* pathogenesis. The growth of the d-alanine auxotroph is dependent on the availability of crucial metabolites in the host tissues. The double mutant auxotroph of *S. aureus* hence showed increased pathogenic loads in host cytosol due to d-alanine supplementation during the course of infection [78]. However, removal of d-alanine restored the attenuated profile of the auxotroph which was also observed in our study. Similar model could be replicated in vivo for modulating the virulence phenotype of the d-alanine auxotroph causing transient pathogenic survival for induction of strong immune responses. An auxotroph of *Listeria monocytogenes* was observed to lack immunogenic potential without d-alanine supplementation hence the vaccination strategy required the addition of the bacteria together with d-alanine [48]. Hence an urgency for vaccine production is vital by utilizing modified immunization strategy against these microbial threats.

## Conclusion

Our findings demonstrated a significant contribution of SEN3897 in vitro and in vivo for invasion and intracellular survival due to possible modulation of SPI-1 and SPI-2 effectors. Identification of novel virulence factors like SEN3897 associated with *S*. Enteritidis hence would aid in the development of potential therapeutics to combat non-typhoidal *Salmonella* infections. *Salmonella* displays an excellent example of regulated and coordinated expression of virulence factors contributing to its pathogenesis. This complex network involves genes central to metabolism, cell wall maintenance, quorum sensing, biofilm development and global regulation for establishment of infection through its virulence machinery. Thus *Salmonella* infections initiate in the intestinal lumen and then disseminate to systemic sites; hence insights into new regulatory networks and identification of novel virulence factors will help discover unique antibacterial drugs for infection control.

## Additional files

**Additional file 1: Table S1.** Primer list used in this study.

**Additional file 2: Figure S1.** PCR screening of alanine racemase gene inactivation. (A) Confirmation of PCR mediated gene replacement of *alr*, *dadX* and SEN *∆3897* in *Salmonella* Enteritidis by kanamycin cassette with internal kanamycin specific primers and confirmatory primers (B) PCR confirmation of double deletions (SEN*∆alr∆dadX*) in *S*. Enteritidis by phage transduction with FRT specific primers for kanamycin and chloramphenicol cassettes of 1.5 kb and 1.1 kb size respectively. These ethidium bromide stained gels were analyzed by agarose gel electrophoresis. The 1 kb DNA ladder (Invitrogen) with the desired product sizes is indicated with arrows.

**Additional file 3: Figure S2.** Growth curve experiment (cfu/mL) to check the cell viability in SEN *∆alr*, SEN *∆dadX*, SEN *∆3897*, WT *Salmonella* Enteritidis and *alr dadX* double mutant in liquid culture with d-alanine supplementation (5 µg/ml, 10 µg/ml, and 50 µg/ml) at different time intervals. Data is represented as mean ± SD.

**Additional file 4: Figure S3.** (A) Sequence alignment of three alanine racemases from *S*. Enteritidis (Alr, DadX, SEN3897). Alr (SEN4016) showed 91% with Y274f Alanine Racemase from *E. Coli* (PDB-ID-4WR3), DadX (SEN1235) showed 48% identity with Alanine Racemase from *Pseudomonas aeruginosa*, (PDB-ID-1RCQ) and SEN3897 showed 42% identity with Chain A, Alanine Racemase from *Bartonella henselae* (PDB-ID-3KW3). (B) Ribbon protein structures determined by I-TASSER. Superimposed alanine racemase structures of Alr (green), DadX (red) and SEN3897 (turquoise Blue) denote their structural similarities with residue conservation.

**Additional file 5: Table S2.** Active site residues of alanine racemase proteins (Alr, DadX and SEN3897) of *Salmonella* Enteritidis from CASTp analysis.

**Additional file 6: Figure S4.** Expression of SEN3897 in WT and SEN *∆alr∆dadX* bacterial strains through qRT-PCR analysis. All experiments were performed in triplicates with data represented as mean ± SD. Statistical significance: **P < 0.01.

**Additional file 7: Figure S5.** Adhesion assay of SEN *∆alr*, SEN*∆dadX*, SEN *∆3897* and WT in HCT116 cell-lines for 30 min at MOI 10. Statistical significance: *P < 0.05.

**Additional file 8: Figure S6.** Survival Assay of SEN *∆alr∆dadX*, Alr complemented strain (pCH112-*alr* in SEN *∆alr∆dadX*) and WT in RAW264.7 in murine macrophages at 24 h time-point. Statistical significance: *P < 0.05, **P < 0.01, ***P < 0.001, ****P < 0.0001.

**Additional file 9: Figure S7.** Fluorescence microscopy images of murine macrophages RAW264.7 infected with SEN *∆alr*, SEN *∆dadX*, SEN *∆3897* and WT for 1 h at MOI 10, gentamicin treated for 24 h and fixed. Cell nuclei were stained with DAPI (blue) and *Salmonella* were GFP-tagged (green). Scale bar: 10 μm.

**Additional file 10: Figure S8.** Colonization characteristics of SEN *∆alr*, SEN *∆dadX*, SEN *∆3897*, SEN *∆alr∆dadX* and WT: Feces from each group of 5 mice were collected and suitable dilutions (1:200, 1:40,000) were plated on MacConkey agar plates with required antibiotics at 24 h and 48 h p.i for assessing the bacterial counts. Statistical significance: *P < 0.05, **P < 0.01.

**Additional file 11: Figure S9.** (A) Invasion assay of SEN *∆alr∆3897* and WT in HCT116 cell-lines. SEN *∆invC* acts as the negative control for invasion assay (B) Uptake Assay and (C) Survival Assay of SEN *∆alr∆3897* and WT in RAW264.7 in murine macrophages at 2 h and 24 h time-points respectively. Statistical significance: *P < 0.05, ****P < 0.0001.

## Abbreviations

WT: wild type
SEN: *Salmonella* Enteritidis
SEN *Δalr*: *alr* deletion mutant of *Salmonella* Enteritidis
SEN *ΔdadX*: *dadX* deletion mutant of *Salmonella* Enteritidis
SEN*Δ3897*: *SEN3897* deletion mutant of *Salmonella* Enteritidis
SEN *ΔalrΔdadX*: *alr* and *dadX* double deletion mutant of *Salmonella* Enteritidis
MOI: multiplicity of infection
CFU: colony forming unit
HCT116: human colon cancer epithelial cells
RAW264.7: murine macrophage cell line
SPI-1: *Salmonella* pathogenicity island 1
SPI-2: *Salmonella* pathogenicity island 2
PBS: phosphate buffered saline
mLN: mesenteric lymph node
