# Supplementary material for: Identification of a new alanine racemase in Salmonella Enteritidis and its contribution to pathogenesis
Source: Gut Pathog. 2018 Jul 10;10:30. doi: 10.1186/s13099-018-0257-6 (PMC6040060; doi:10.1186/s13099-018-0257-6)
Supplement: Supplementary file 1 — Additional file 1: Table S1. Primer list used in this study. [file 13099_2018_257_MOESM1_ESM.doc]

**ADDITIONAL FILE 1**

**Table S1: Primers used in study**

|  |  |  |  |
| --- | --- | --- | --- |
| **Primer** | **Sequence (5’-3’)** | Description | Reference |
| Fw Ko SEN1235 (dadX) | GAAGTGAAATGACCCGCCCTATACAGGCCAGCCTTGATT TACAGGtgt gtaggctggagctgctt | Knockout and confirmatory primers for alanine racemase gene mutation; FRT-kanamycin-FRT amplification | This study |
| Rw Ko SEN1235 (dadX) | GACCCGCCCCCAGGTGGACCGGTCGACGCCTTAGTC TGAGTTAGGatatgaatatcctccttagt |
| Conf Ko SEN1235 (dadX) | TTCCGATTTTACGCCCACCC |
| Fw Ko SEN4016 (alr) | TCTCCGTCATTCTGTTAACAAGGAATTCAAATGCAA GCGGCAACAtgtgtaggctggagctgctt |
| Rw Ko SEN4016 (alr) | GATACAAGCCGGATAAGCGCAGGCGCCACCCGGCCC GCCGCGTATatatgaatatcctccttagt |
| Conf Ko SEN4016 (alr) | CTATGCGGGACCGCAATACG |
| Fw Ko SEN3897 | GAAACTTGCTGAAATTCAGGCGGCGTGCGGCGTT CTTTGTGTAGAtgtgtaggctggagctgctt |
| Rw Ko SEN3897 | CTCATAAGGGATGGTATCGCAGGCGGCGGC AACCTCTTCCAGCGTatatgaatatcctccttagt |
| Conf Ko SEN3897 | GCCATCAAGACGCCGAATATTACAG |
| Km/Kt | CGGTCCGCCACACCCAGCC |
| Fw sseJ | CCGGAAGCTTTTGGTCTTGC | SPI-2 effectors |
| Rw sseJ | GCCGATGTACTTCCCCCTTC |
| Fw sseG | GGTCATTGCCATCCCATCCA |
| Rw sseG | TTGCCTATGGCTCACGACAG |
| Fw SipA | TCCAGTTGCGGGCCTTTAAT | SPI-1 effector | [1] |
| Rw SipA | GCAGACCGAGATCAAAACGC |
| Fw InvF | TCCACTAATCCTGCGCCATC | SPI-1 Regulators |
| Rw InvF | CCGTTGTCGCACCAGTATCA |
| Fw HilA | GGGCAGATGATACCCGATGG |
| Rw HilA | AAGAGAGAAGCGGGTTGGTG |
| Fw HilD | TGACAAATACCCGGCAGCAA |
| Rw HilD | AGTTTACCGCTCCGAAAGCA |
| Fw H-NS | ACGAGTGCGTTCTTCCACTT | DNA-binding Transcriptional repressor | This study |
| Rw H-NS | ACATCCGTACTCTTCGTGCG |
| Fw 16s rRNA | TTCCAGTGTGGCTGGTCATC | Housekeeping gene |
| Rw 16s rRNA | TGCCTGATGGAGGGGGATAA |
| Fw pET NdeI dadX | CAATTTCATATGATGACCCGCCCTATACAGGCC | Cloning primers for protein expression | This study |
| Rw pET XhoI dadX | CAATTTCTCGAGTTACGTTGTCACAAATGGCACGC |
| Fw pET NdeI alr | CAATTTCATATGATGCAAGCGGCAACAGTCGTC |
| Rw pET XhoI alr | CAATTTCTCGAGTTAATCAATATACTTCATCGCCACCCTTG |
| Fw pET NdeI 3897 | CAATTTCATATGATGATGAAACTTGCTGAAATTCAGGC |
| Rw pET XhoI 3897 | AAATTTCTCGAGTCAAGCTTCTGCGGGCTGC |
| Fw SEN3897 | CCAATGGCTATGGTCTGGGG | Real-time primers for expression studies | This study |
| Rw SEN3897 | AACAGAATTCAGCAACGGCG |
| Fw SENalr | AGCGGCAACAGTCGTCATTA |
| Rw SENalr | AAAAGCGTCAGCATCAGGGA |
| Fw SENdadX | GCGTCTGGTCGGTAGTGAAA |
| Rw SENdadX | TCCAGCATCAATATCGGCCC |
| Fw pCH Alr NcoI | CAGATCCCATGGATGGGTAAAACCACTTTTGCGATG | Cloning primers for *alr* complementation |
| Rw pCH Alr XbaI | CAGATCTCTAGATTAATCAATATACTTCATCGCCACCCTTG |

**REFERENCES**

1. Ryan D, Ojha UK, Jaiswal S, Padhi C, Suar M. The small RNA DsrA influences the acid tolerance response and virulence of *Salmonella enterica* serovar Typhimurium. Front Microbiol. 2016;7:599
