# Supplementary figures and images for: Identification of a new alanine racemase in Salmonella Enteritidis and its contribution to pathogenesis
Source: Gut Pathog. 2018 Jul 10;10:30. doi: 10.1186/s13099-018-0257-6 (PMC6040060; doi:10.1186/s13099-018-0257-6)

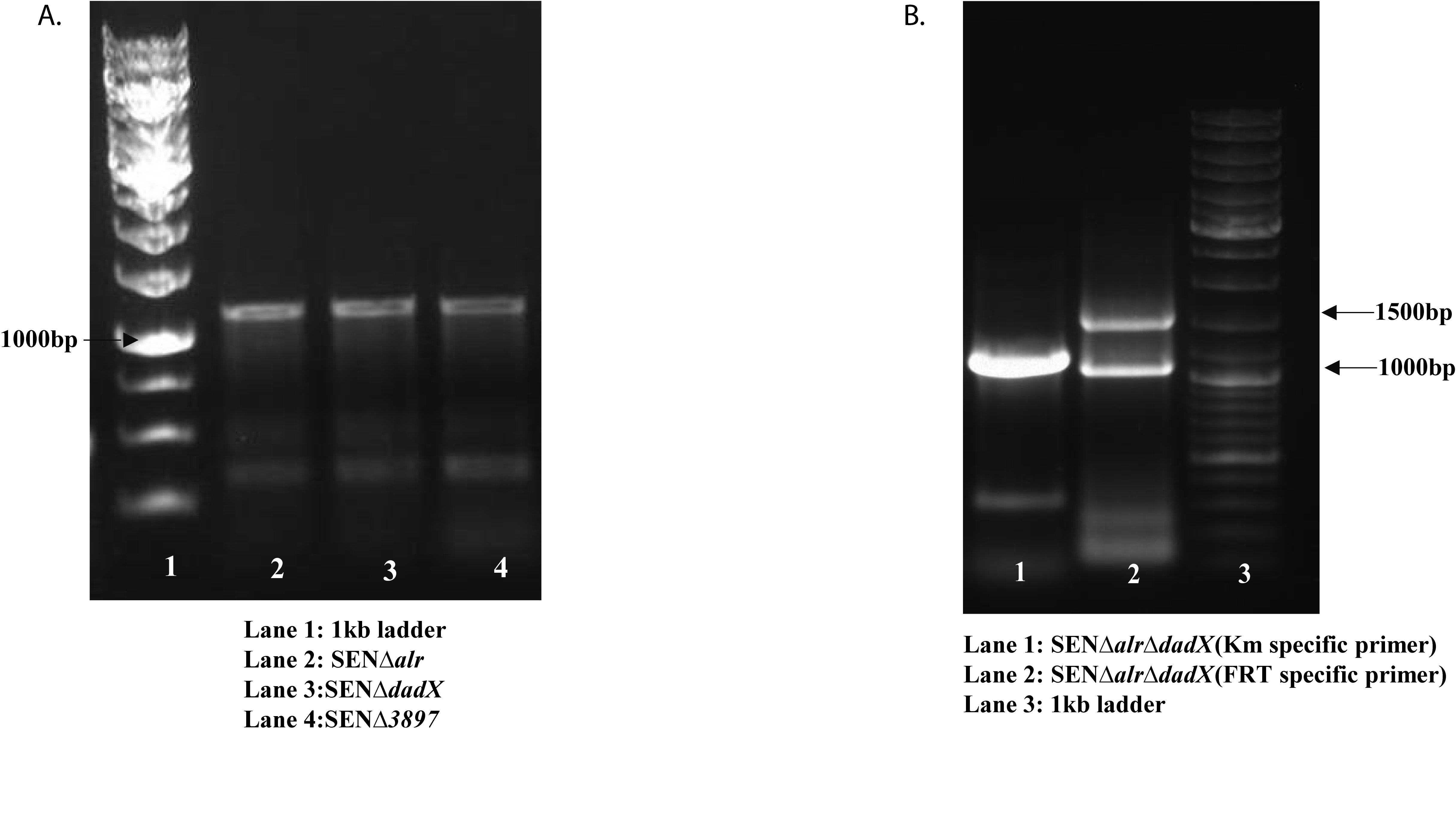

Supplement: Supplementary file 2 — Additional file 2: Figure S1. PCR screening of alanine racemase gene inactivation. (A) Confirmation of PCR mediated gene replacement of alr, dadX and SEN ∆3897 in Salmonella Enteritidis by kanamycin cassette with internal kanamycin specific primers and confirmatory primers (B) PCR confirmation of double deletions (SEN∆alr∆dadX) in S. Enteritidis by phage transduction with FRT specific primers for kanamycin and chloramphenicol cassettes of 1.5 kb and 1.1 kb size respectively. These ethidium bromide stained gels were analyzed by agarose gel electrophoresis. The 1 kb DNA ladder (Invitrogen) with the desired product sizes is indicated with arrows. [file 13099_2018_257_MOESM2_ESM.tif]

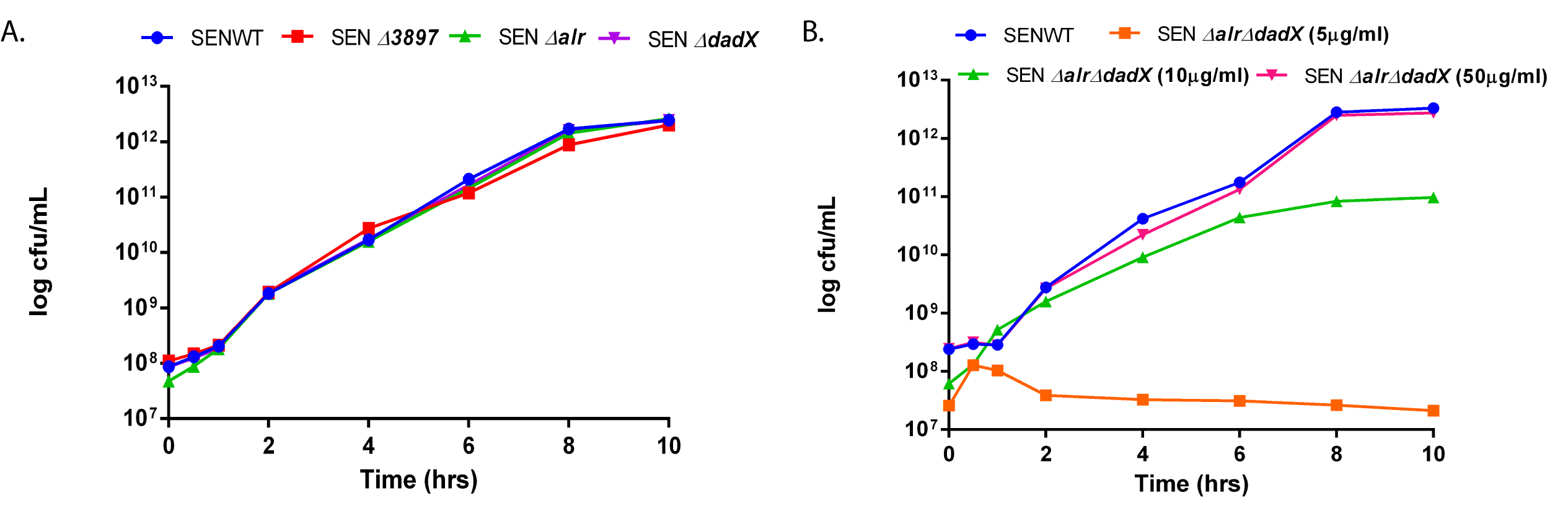

Supplement: Supplementary file 3 — Additional file 3: Figure S2. Growth curve experiment (cfu/mL) to check the cell viability in SEN ∆alr, SEN ∆dadX, SEN ∆3897, WT Salmonella Enteritidis and alr dadX double mutant in liquid culture with d-alanine supplementation (5 µg/ml, 10 µg/ml, and 50 µg/ml) at different time intervals. Data is represented as mean ± SD. [file 13099_2018_257_MOESM3_ESM.tif]

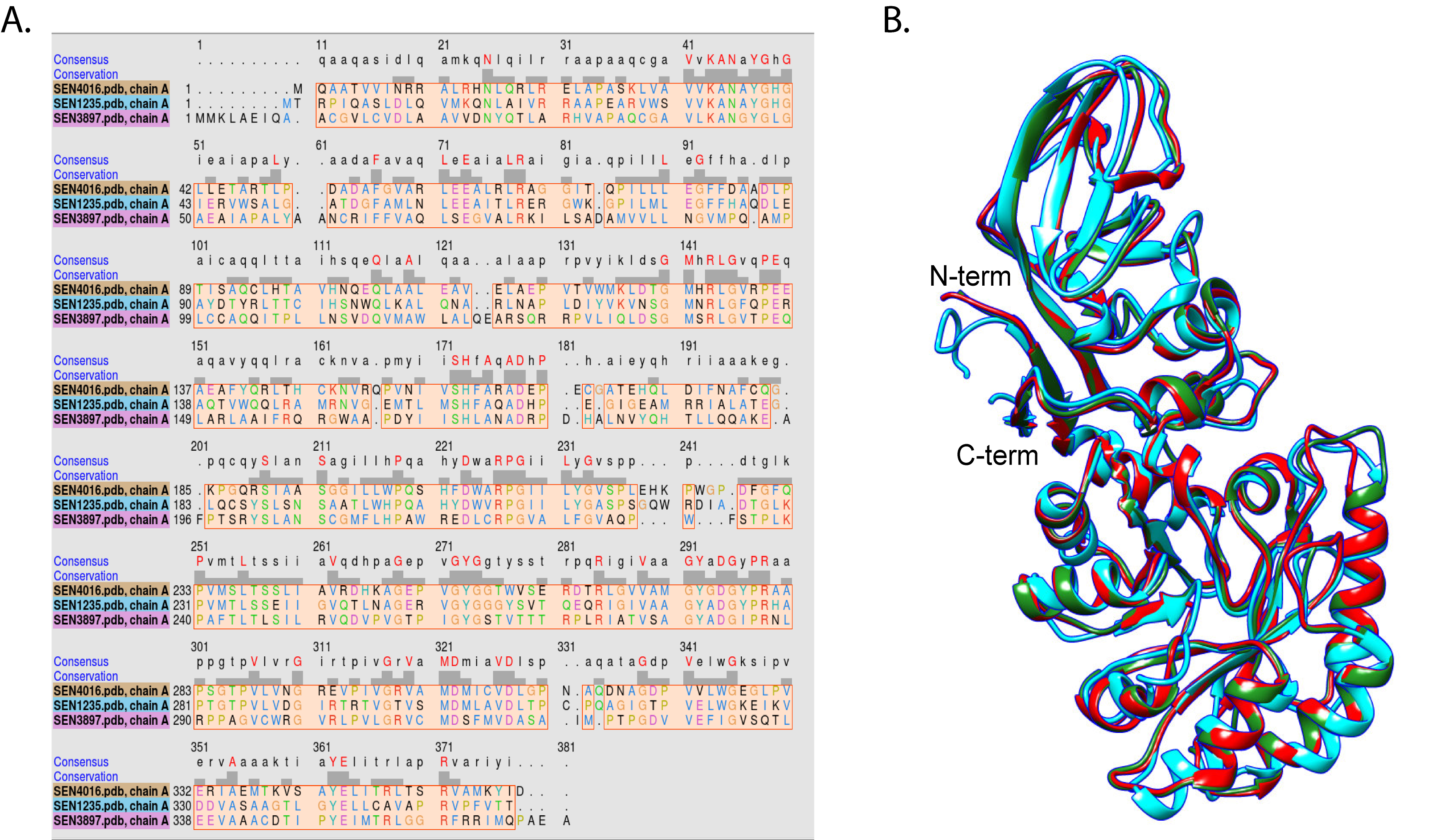

Supplement: Supplementary file 4 — Additional file 4: Figure S3. (A) Sequence alignment of three alanine racemases from S. Enteritidis (Alr, DadX, SEN3897). Alr (SEN4016) showed 91% with Y274f Alanine Racemase from E. Coli (PDB-ID-4WR3), DadX (SEN1235) showed 48% identity with Alanine Racemase from Pseudomonas aeruginosa, (PDB-ID-1RCQ) and SEN3897 showed 42% identity with Chain A, Alanine Racemase from Bartonella henselae (PDB-ID-3KW3). (B) Ribbon protein structures determined by I-TASSER. Superimposed alanine racemase structures of Alr (green), DadX (red) and SEN3897 (turquoise Blue) denote their structural similarities with residue conservation. [file 13099_2018_257_MOESM4_ESM.tif]

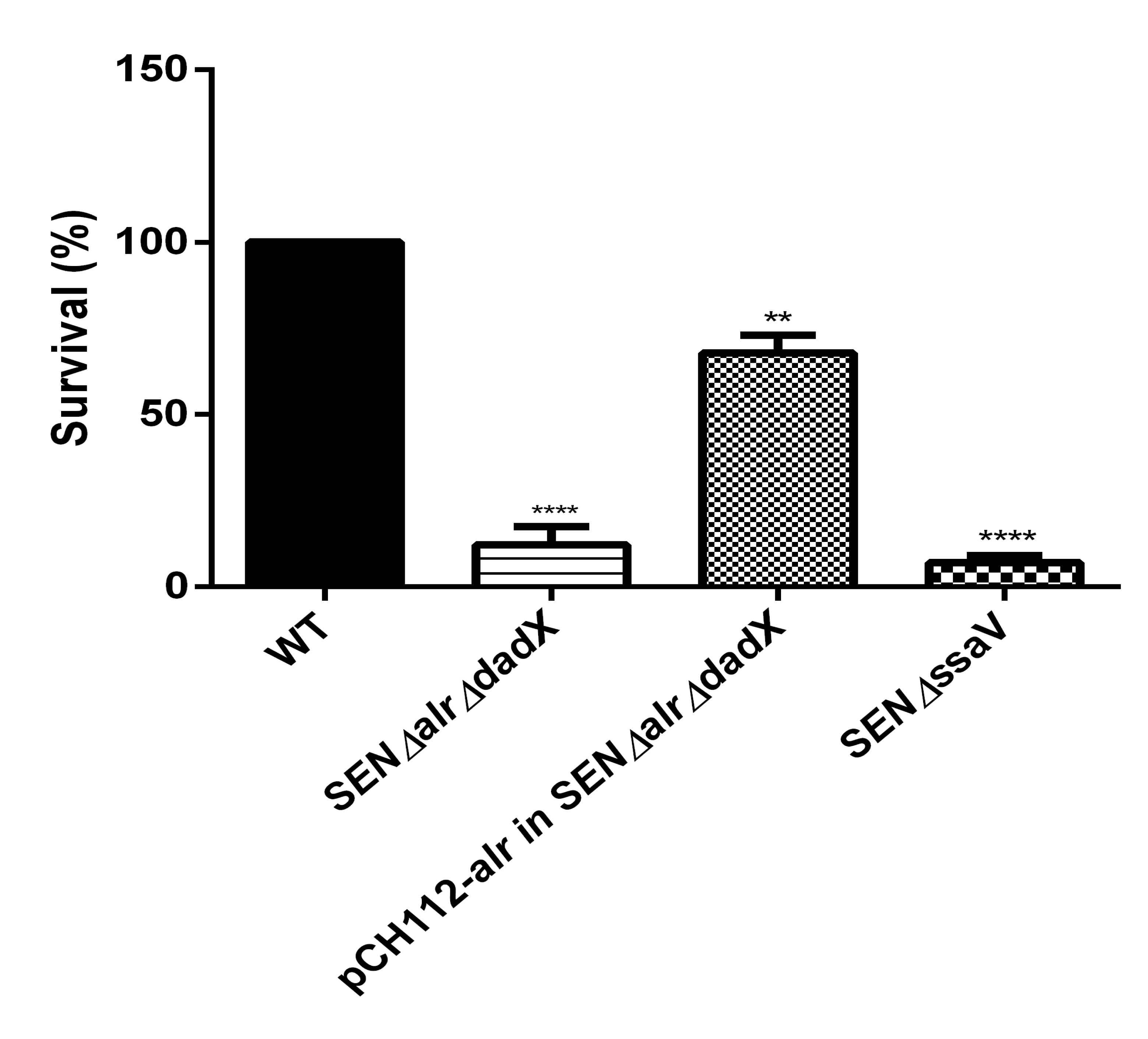

Supplement: Supplementary file 8 — Additional file 8: Figure S6. Survival Assay of SEN ∆alr∆dadX, Alr complemented strain (pCH112-alr in SEN ∆alr∆dadX) and WT in RAW264.7 in murine macrophages at 24 h time-point. Statistical significance: *P < 0.05, **P < 0.01, ***P < 0.001, ****P < 0.0001. [file 13099_2018_257_MOESM8_ESM.tif]

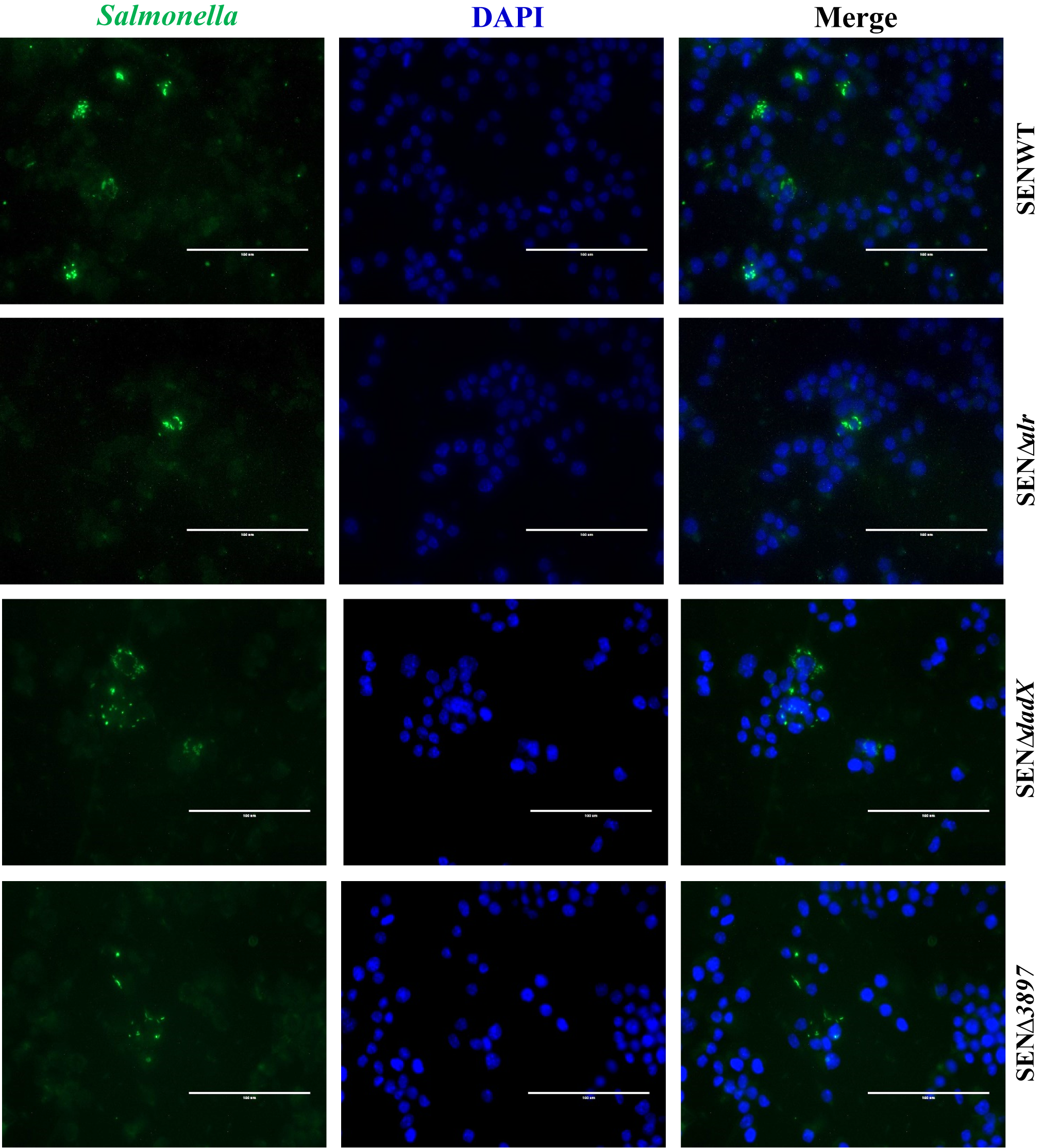

Supplement: Supplementary file 9 — Additional file 9: Figure S7. Fluorescence microscopy images of murine macrophages RAW264.7 infected with SEN ∆alr, SEN ∆dadX, SEN ∆3897 and WT for 1 h at MOI 10, gentamicin treated for 24 h and fixed. Cell nuclei were stained with DAPI (blue) and Salmonella were GFP-tagged (green). Scale bar: 10 μm. [file 13099_2018_257_MOESM9_ESM.tif]

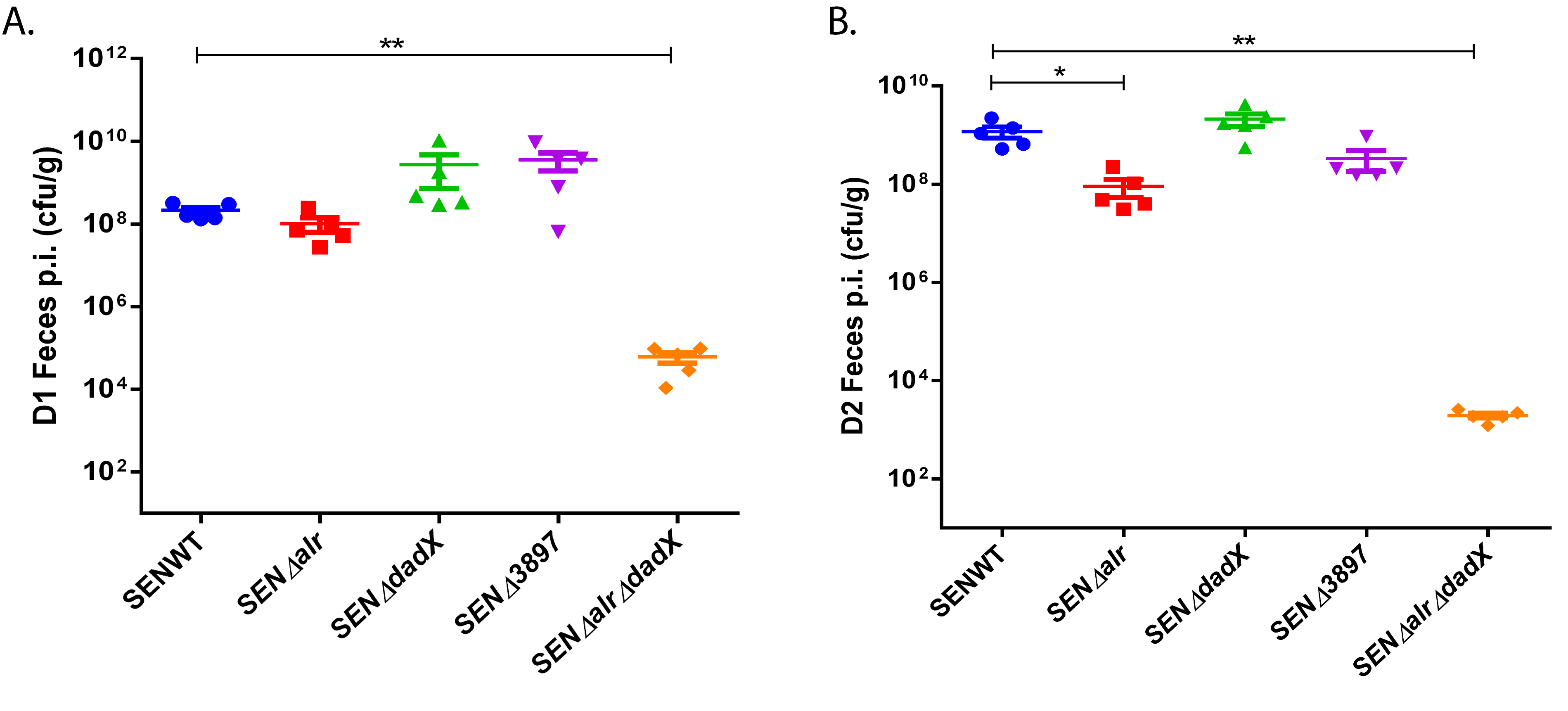

Supplement: Supplementary file 10 — Additional file 10: Figure S8. Colonization characteristics of SEN ∆alr, SEN ∆dadX, SEN ∆3897, SEN ∆alr∆dadX and WT: Feces from each group of 5 mice were collected and suitable dilutions (1:200, 1:40,000) were plated on MacConkey agar plates with required antibiotics at 24 h and 48 h p.i for assessing the bacterial counts. Statistical significance: *P < 0.05, **P < 0.01. [file 13099_2018_257_MOESM10_ESM.tif]

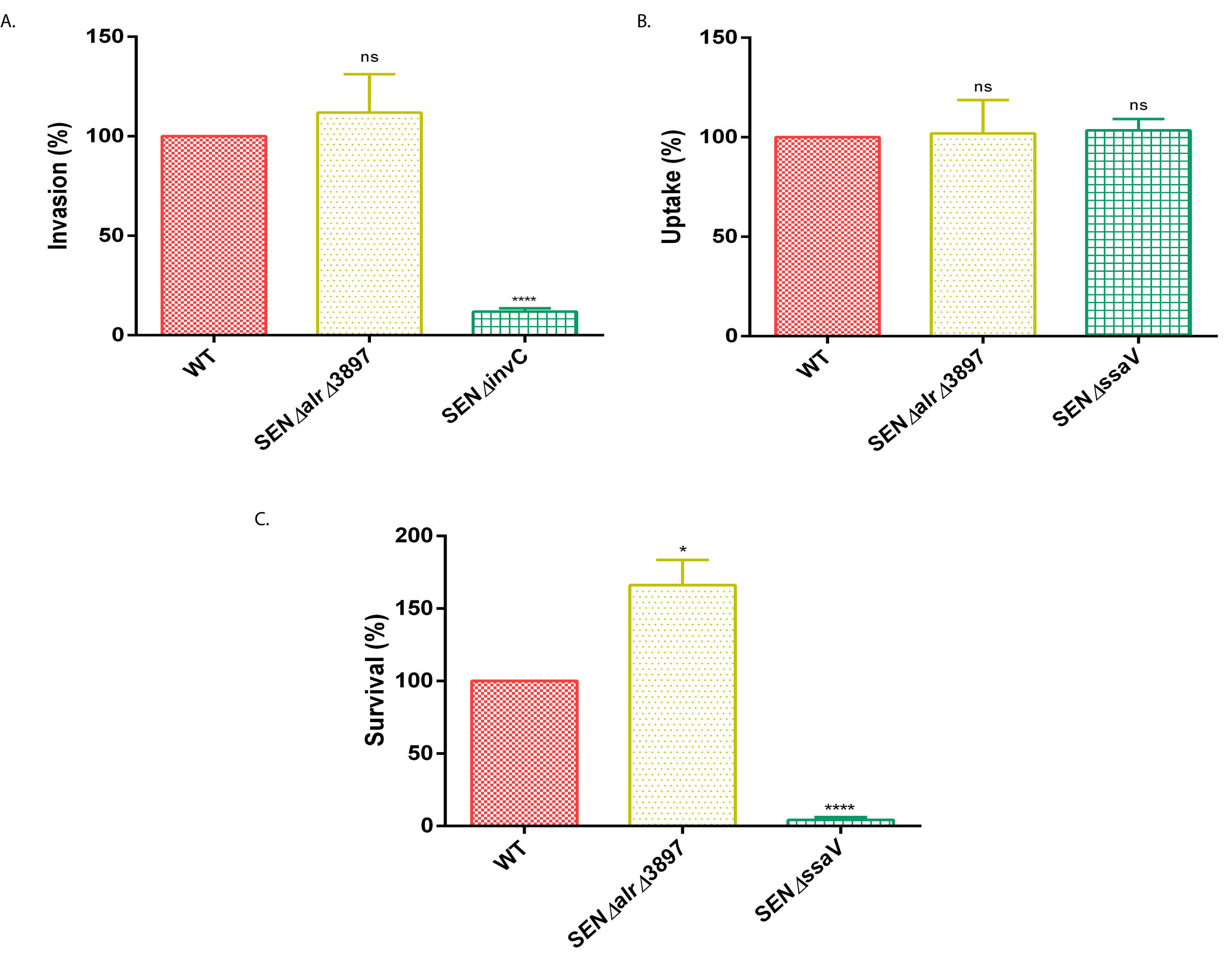

Supplement: Supplementary file 11 — Additional file 11: Figure S9. (A) Invasion assay of SEN ∆alr∆3897 and WT in HCT116 cell-lines. SEN ∆invC acts as the negative control for invasion assay (B) Uptake Assay and (C) Survival Assay of SEN ∆alr∆3897 and WT in RAW264.7 in murine macrophages at 2 h and 24 h time-points respectively. Statistical significance: *P < 0.05, ****P < 0.0001. [file 13099_2018_257_MOESM11_ESM.tif]
