# Supplementary material for: Identification of a new alanine racemase in Salmonella Enteritidis and its contribution to pathogenesis
Source: Gut Pathog. 2018 Jul 10;10:30. doi: 10.1186/s13099-018-0257-6 (PMC6040060; doi:10.1186/s13099-018-0257-6)
Supplement: Supplementary file 5 — Additional file 5: Table S2. Active site residues of alanine racemase proteins (Alr, DadX and SEN3897) of Salmonella Enteritidis from CASTp analysis. [file 13099_2018_257_MOESM5_ESM.doc]

**Additional file 5**

**Table S2: Active site residues of alanine racemase proteins from CASTp analysis**

| **Structures** | **Active sites** |
| --- | --- |
| **SEN4016 (Alr)** | VAL32, **LYS34**, TYR38, ALA58, LEU76, LEU78, GLU79, ALA98, LYS122, LEU123, ASP124, THR125, MET127, ARG129, LEU130, VAL157, SER158, HIS159, PHE160, ALA163, ASP164, ILE191, ALA193, SER194, GLY195, ARG209, GLY211, ILE212, PRO219, **TYR343** |
| **SEN3897** | VAL40, **LYS42**, TYR46, PHE66, VAL67, ALA68, LEU89, LEU108, GLN134, LEU135, ASP136, SER137, MET139, ARG141, LEU142, SER169, HIS170, LEU171, ALA172, ALA174, ASP175, ARG176, ASN205, SER206, CYS207, ARG221, GLY223, VAL224, VAL229, ALA230, GLN231, ILE347, PRO348, **TYR349** |
| **SEN1235 (DadX)** | VAL33, LEU59, LEU79, GLU80, LYS123, VAL124, ASN125, SER126, MET128, ARG130, LEU131, MET157, SER158, HIS159, PHE160, ALA161, GLN162, ALA163, ASP164, HIS165, ASN191, SER192, ALA193, ARG207, GLY209, ILE210, ALA215, SER216, PRO217, SER218, GLY219, TRP221, ARG222, LYS328, VAL329, ASP330, ALA333, SER334, THR338, LEU339, GLY340, **TYR341** |
